# Nanotechnology-Based Topical Delivery of Natural Products for the Management of Atopic Dermatitis

**DOI:** 10.3390/pharmaceutics15061724

**Published:** 2023-06-14

**Authors:** Mário Pedro Marques, Carla Varela, Laura Mendonça, Célia Cabral

**Affiliations:** 1 Coimbra Institute for Clinical and Biomedical Research (iCBR), Clinic Academic Center of Coimbra (CACC), Faculty of Medicine, University of Coimbra, 3000-548 Coimbra, Portugal; mpedromarques@outlook.pt (M.P.M.); carlalvarela@gmail.com (C.V.); laurafmendonca@gmail.com (L.M.); 2 Center for Innovative Biomedicine and Biotechnology (CIBB), University of Coimbra, 3000-548 Coimbra, Portugal; 3 Chemical Process Engineering and Forest Products (CIEPQPF), Faculty of Medicine, University of Coimbra, 3000-548 Coimbra, Portugal; 4 Centre for Functional Ecology, Department of Life Sciences, University of Coimbra, 3000-548 Coimbra, Portugal

**Keywords:** atopic dermatitis, skin inflammation, natural products, nanotechnology, topical delivery

## Abstract

Atopic dermatitis (AD) is a chronic eczematous inflammatory disease that may arise from environmental, genetic, and immunological factors. Despite the efficacy of current treatment options such as corticosteroids, such approaches are mainly focused on symptom relief and may present certain undesirable side effects. In recent years, isolated natural compounds, oils, mixtures, and/or extracts have gained scientific attention because of their high efficiency and moderate to low toxicity. Despite their promising therapeutic effects, the applicability of such natural healthcare solutions is somewhat limited by their instability, poor solubility, and low bioavailability. Therefore, novel nanoformulation-based systems have been designed to overcome these limitations, thus enhancing the therapeutic potential, by promoting the capacity of these natural drugs to properly exert their action in AD-like skin lesions. To the best of our knowledge, this is the first literature review that has focused on summarizing recent nanoformulation-based solutions loaded with natural ingredients, specifically for the management of AD. We suggest that future studies should focus on robust clinical trials that may confirm the safety and effectiveness of such natural-based nanosystems, thus paving the way for more reliable AD treatments.

## 1. Introduction

The skin is the largest human organ, covering an area of approximately 1.8 m^2^, comprising three main layers: the epidermis as the outermost layer, the dermis, and the hypodermis. Skin protects the human body from several external harmful agents, reduces electrolyte loss, and regulates evapotranspiration and body temperature, plus it consists of an immune defense barrier against microorganisms [1]. It is estimated that half of the adult population have developed some kind of skin ailment at one point in their lives, with 1/3 manifesting chronic or mild skin diseases. In fact, skin diseases represent a major concern affecting the quality of life of children, teenagers, and adults [2].

AD, also known as eczema, is a chronic relapsing inflammatory skin disorder, characterized by recurrent eczematous lesions and severe skin itching. This inflammatory skin disease tends to appear mostly in the first five years of age, thus affecting nearly 30% of children and teenagers, in comparison to only 2–10% of adults. However, it is currently assumed that AD may appear at any age. Besides the mental impact on patients with increasing probability of depression and suicide, plus severe skin itching and pain, there are also many associated costs, including skin cleaning products, appropriate clothes, creams, and ointments. Interestingly, the real pathogenic source of the disease is not yet fully comprehended, but the main findings point to the interaction between three major mechanisms, that are skin structure defects, changes in the skin microbiome, and impairment of Th2 immune responses [3,4,5,6].

Nowadays, the clinical treatment for AD relies on topical application of corticosteroids, topical calcineurin inhibitors, antihistamines, antibiotics, systemic immunosuppressors, and phototherapy. Despite the efficacy of such treatments, the derived side effects of such approaches are an equally challenging reality. Among these undesired therapy outcomes, skin atrophy, striae, telangiectases, rosacea and acne, glaucoma, hyperglycemia, and hypertension are some examples of problems coming from corticosteroids’ topical application [7,8,9]. On the contrary, natural products and derived isolated bioactive compounds are emerging alternatives to these synthetic drugs, given their high efficacy rates while reducing side effects, plus the greater cost-effectiveness [2]. Indeed, medicinal plants comprise the primary healthcare solutions of nearly 65% of the worldwide population, and almost 80% of people inhabiting developing countries [1].

Natural products are the source of several, and highly heterogenous, molecules such as multiple phenolic ring-bearing compounds such as flavonoids, tannins, and catechins, nitrogen-containing molecules such as alkaloids, carotenoids, and polysaccharides, and small volatile molecules such as those found in essential oil-bearing plants [2]. Interestingly, their anti-inflammatory properties in the treatment of skin inflammation-based diseases have been depicted, such as for vitiligo [10], psoriasis [11], and, more importantly, AD [9]. Recent research suggest that these molecules exert an antioxidant activity, improving cells’ redox status, that in turn ameliorates the inflammatory response, by suppressing the activity of key regulators in mitogen-activated protein kinase (MAPK) and nuclear factor kappa-light-chain-enhancer of activated B cells (NF-κB) signaling pathways, which are key molecular inflammatory responses [8]. To overcome some transdermal delivery problems, augment drug-to-site targeting, solve problems related to active compounds’ solubility, and increase efficacy of such natural ingredients in AD treatment, some nanotechnology-based solutions have been developed in recent years. As successful examples that are herein reviewed, investigators designed quercetin nanostructured lipid carriers [12], solid lipid nanoparticles loaded with capsaicin, curcurmin, and resveratrol [13], nanoparticles of epigallocatechin-3-gallate [14,15], transfersomes loaded with glycyrrhizic acid [16], innovative phytosomes with *Centella asiatica* (L.) Urban extracts [17], nanocapsule-based films of pomegranate seed oil [18], and ethosome-based cream of tea tree oil [19], and several other formulations presented throughout this paper.

**Figure 1:**
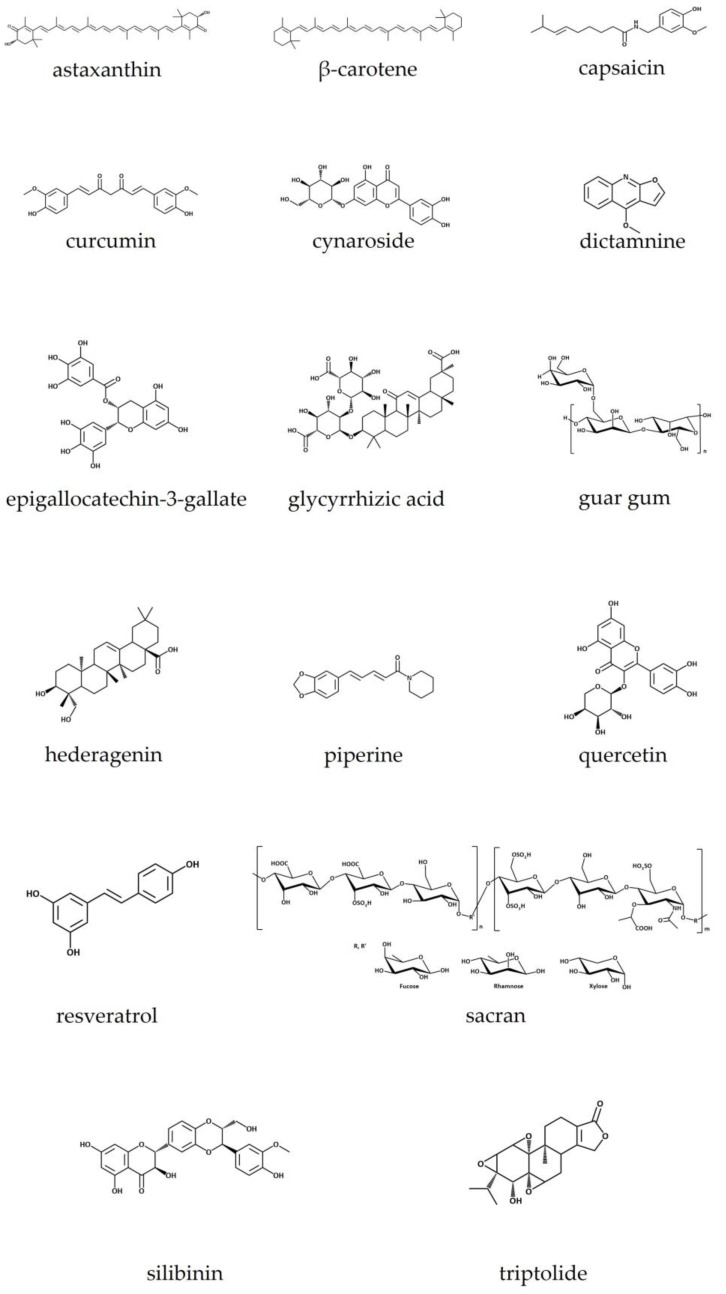
Chemical structures of relevant isolated natural compounds included in nanoformulations for the management of AD. Chemical structures were drawn using ChemDraw software.

**Figure 2:**
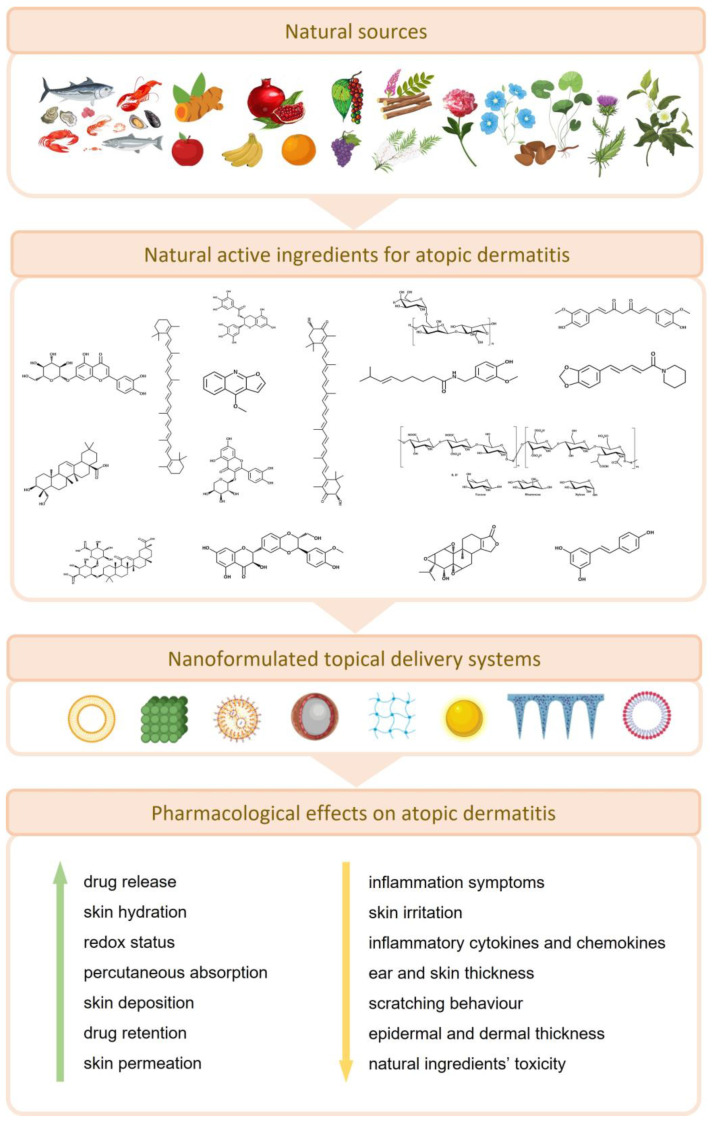
Schematic illustration of isolated natural ingredients included in different nanosystem types for improved pharmacological effect on AD management.

In this review, we focused on natural isolated compounds and plant-based extracts/mixtures and oils that have been included in recent years in nanotechnology-based formulations for the treatment of AD. Some background information about the disease pathophysiology, and nanotechnology tools available to treat it, is provided for a comprehensive interpretation of the topics herein included. Besides that, for each natural product and nanosystem herein reviewed, some pharmacological activity insights, natural products’ physicochemical features, and major natural sources are summarized. As far as we know, this is the first review that has focused on describing the most recent and innovative nanotechnology-based formulations loaded with natural products for AD treatment. Therefore, information about nanotechnology-based formulations loaded with natural isolated compounds is reviewed in Section 5, with key information summarized in Table 1 and Table 2, as well as in Figure 1 and by the graphical abstract provided in Figure 2. Similarly, the extracts, oils, and plant mixtures are presented in Section 6, with relevant information on this topic gathered in Table 3 and Table 4.

## 2. Materials and Methods

A comprehensive analysis of upcoming surveys regarding the application of nanotechnology-based formulations for the delivery of natural products for AD treatment was carried out. In this review, an extensive review of the literature was carried out regarding a span of ten years, from 2013 to 2023. The search was performed in databases such as ScienceDirect, Scopus, PubMed, Web of Science, and Google Scholar. The following keywords were applied individually and/or in combination: atopic dermatitis, eczema, inflammation, skin, natural products, natural compounds, alkaloids, phenolic compounds, flavonoids, terpenes, polysaccharides, oils, plant extract, drug delivery, nanosystems, formulation, nanotechnology. After screening the literature, 16 natural isolated compounds and 8 plant-derived extracts/mixtures and oils were investigated in depth for their pharmacological activity on AD and respective nanoformulation-based drug delivery systems that have been recently reported.

## 3. Pathophysiology and Clinical Treatment Approaches of AD

Among inflammatory skin disorders, AD is one of the most common, affecting between 15 and 30% of children and up to 10% of adults in high-income countries [3,4,5,6]. Atopic eczema and eczema are other names attributed to this condition, being interchangeable terms [3]. AD is related to a null mutation in the filaggrin gene which compromises the *stratum corneum*, changing the epidermal barrier function. This dysfunction leads to an increased exposure to external irritants and allergens [4]. This condition is characterized by itch and pain during flares, leading to a huge impact on the patient’s life, affecting growth, mental health, and work productivity and leading to other burdens such monetary ones [3].

Atopy, the tendency to produce an exaggerated immunoglobulin E (IgE) immune response, is characteristic of AD, asthma, and allergic rhinitis, thus individuals that have AD usually also have associated conditions [6]. There is no direct test to diagnose AD, so it is confused with other similar skin conditions such as psoriasis and keratosis pilaris, with a complex diagnostic that may take years to be precise. In newborns AD usually manifests first during teething [4].

A multidisciplinary approach is required to properly manage and treat AD. As children are the most affected, family education on this condition and especially how to prevent flares is important [3]. Recording the family history related to AD incidence is of the greatest importance, because genetics and the environment are decisive factors in this condition. Treatments aim fundamentally at restoring the skin barrier and controlling abnormal immune responses. The first step in prevention is to avoid irritants and allergens [6].

Regarding clinical therapeutics, there are two major options for AD treatment: systemic and non-systemic therapeutics. Current systemic therapeutic options include biologics (dupilumab, lebrikizumab, nemolizumab, omalizumab, and tralokinumab), conventional immunosuppressive drugs (azathioprine (AZA), ciclosporin (CyA), glucocorticosteroids, methotrexate (MTX), and mycophenolate mofetil (MMF)), and Janus kinase (JAK) inhibitors (abrocitinib, baricitinib, and upadacitinib) [41]. To date, broad-acting immunosuppressants, such as CyA, AZA, MMF, enteric coated mycophenolate sodium (EC-MPS), and MTX are systemic treatments used for severe AD cases and can be classified in two different groups. The first group comprises synthetic drugs such as CyA, which act rapidly and can be used to treat flares of AD. The pharmacokinetics of JAK inhibitors baricitinib, abrocitinib, and upadacitinib classify this group as having a fast action onset. On the other hand, the Th2-blocking agents dupilumab, tralokinumab, and lebrikizumab, as well as the IL31-receptor-blocking agent nemolizumab, require more time to reach the desired efficacy [41]. As an example, in severe cases, immunosuppressants such as anti- interleukin-4 (IL-4) are a very efficient option that changed how AD is treated, but should be avoided in long-term treatments [6].

In turn, non-systemic therapeutic options comprise mainly emollients and moisturizers, such as those with non-medical active ingredients, cleansing and bathing procedures, topical corticosteroids, topical phosphodiesterase 4 inhibitors, topical antimicrobial treatment, improved dietary habits, complementary medicine practices, etc. [42]. For example, the use of emollients twice a day hugely reduces the probability of flares. Meanwhile, treatment with topical corticosteroids should be considered only as a last option, especially in children, despite being safe under medical prescription [6].

## 4. Nanotechnology: Safety Issues, Advantages, and Disadvantages of Application

A significant number of drugs display low drug absorption due to different solubilities within body fluids and systems, which results in impaired bioavailability and efficacy in delivery. Nanoformulations have been found to overcome drugs’ poor absorption given their capacity for the desired physicochemical properties. They can have drugs entrapped, encapsulated, dissolved, or linked to their matrix. Their small size, ranging from 10–1000 nm [43], is responsible for their larger specific surface area, which allows an easier administration which can be intranasal, ocular, oral, or subcutaneous. Additionally, they can be designed in a way that can bypass the body’s clearance mechanisms and at the same time target a specific site. Another important advantage of these systems is that they efficiently entrap drugs, protecting them from premature decomposition [44,45]. Other benefits of the use of these nanosystems include the reduction of total dose used and the potential toxic side effects, with better safety and efficacy, and also a drug release at a constant rate with accumulation in desired tissues [46].

Despite the interest in and identified advantages of these products as drug delivery systems, they also have some disadvantages such as tendency to agglomerate, some are not biodegradable, high cost for large-scale production, as well as some toxicity concerns [47]. Their potential harmful effects result from the fact that they may interact with biomolecules and spread over all the human body. Some nanoparticles can dissolve easily while others may accumulate, persisting for long periods of time in biological systems [48]. To address these worries, specific tests are required to distinguish drug-loaded and empty nanoproducts [49]. To date, the management of the adverse reactivity of nanoparticles and their safe handling still require further investigation to really understand their interactions with the body [50]. The most frequently used methods to evaluate skin irritation are the 3D skin models EpiSkinTM^®^ and EpiDermTM^®^, followed by 2D in vitro skin models such as the HaCaT human keratinocyte cell line and the BALB/c 3T3 mouse embryo fibroblast cell line, along with the MTT assay, which is a prominent test to evaluate safe concentrations for topical application of nanotechnology [51].

In fact, several nanomedicines have received regulatory approval to be employed in clinical practice. It is mandatory to design suitable safety tests that ensure the biocompatibility and desired activity for such nanosystems. The assumption that nanotechnology is generally safe, could be problematic, since different manufactured nanoparticles are produced from various materials [52]. In the case of solid lipid nanoparticles (SLNs) and nanostructured lipid carriers (NLCs), several components are not necessarily irrelevant or safe. As an example, these nanocarriers usually contain cationic components, derivatives of cholic acid and salts, and various linkers for attachment of targeting moieties or even sugars that may have impact on the immune system [51]. In this context, for example, carbon-based nanoparticles have been shown to be potentially toxic according to several in vitro and in vivo assays, similarly to what has also been reported for metal-based structures such as gold- and silver-based nanosystems. However, there is growing evidence that such harmful effects may arise from factors such as the size and morphology of particles, instead of the bulk material used in nanosystems’ scaffolds. Nevertheless, several authors have agreed that by modifying the surface properties while enhancing the target-to-site specificity of these innovative delivery systems, harmful effects have been successfully bypassed. Meanwhile, the use of biodegradable, well-tolerated, and physiological excipients have shown NLCs and SLNs to be good alternatives for skin therapeutics. Moreover, these kinds of delivery systems have been shown to avoid the potential skin irritation of many compounds, which could be explained by the encapsulation strategy, owing to a reduction in local skin concentration. Furthermore, according to in vitro safety tests, there is evidence that SLNs and NLCs are safe nanosystems at concentrations of <1 mg/mL total lipids [51].

## 5. Isolated Natural Compounds Included in Nanotechnology-Based Formulations for the Treatment of AD

### 5.1. Astaxanthin

#### 5.1.1. Natural Source, Physicochemical Features, and Bioactive Properties

Astaxanthin (C_40_H_52_O_4_) (Figure 1), with a molecular weight of 596.84 g/mol, is a xanthophyll carotenoid found in living organisms such as microalgae, crustaceans, and seafood, but also in yeast, fungi, complex plants, and birds’ feathers. It is a red-colored lipid-soluble compound that gives marine animals their distinctive red–orange color and protects from UV radiation. Astaxanthin has a peculiar structure: a non-polar region in the middle, with a series of thirteen conjugated double bonds, and two polar regions with two ionone rings with hydroxyl (at 3,3′) and keto (at 4,4′) groups. This explains its simultaneous hydrophobic and hydrophilic behavior. It exists in different forms such as optical stereoisomers, geometric isomers, and free or esterified forms and can be complexed with proteins or lipoproteins. The most predominant form in nature is the esterified one [20,53,54,55].

Astaxanthin displays several biological activities with therapeutic potential and health benefits. It has antioxidant, anti-inflammatory, and antiapoptotic activities which are responsible for its therapeutic use in cancer and obesity, for triglyceride and cholesterol, and as an immunomodulator, antidiabetic, hepatoprotective, and neuroprotective agent, with benefits for the human skin [54].

#### 5.1.2. Drug Delivery Systems and Pharmacological Activity

Astaxanthin has proved to be a strong antioxidant that blocks inflammation at the beginning via NF-κB and hinders inflammatory mediators such as interleukin-1β (IL-1β), interleukin-6 (IL-6), and tumor necrosis factor-α (TNF-α). It also inhibits cyclooxygenase-1 (COX-1) and nitric oxide (NO) [20,54]. Its antidermatitis effect was also confirmed via inhibition of other inflammatory markers: inducible nitric oxide synthase (iNOS), cyclooxygenase-2 (COX-2), and IgE [17,55].

Different formulations have been developed to enhance stability and bioavailability of astaxanthin in topical applications which include nanoemulsions (NEs) [56], hydrogels/lipogels [57], liposomes (LIPs) [20], and NLCs [58].

Of the former, only the work of Lee et al. [20] was focused on the evaluation of the developed formulation in AD. Hence, a liposomal formulation containing astaxanthin (L-AST) was prepared, where the conjugation with phospholipid structures improved the low water solubility of the molecule, hence allowing the study of its effect in the prevention of AD by skin inflammation inhibition. This liposomal astaxanthin was prepared by mixing it with phosphatidylcholine in a 1:4 ratio using a MicrofluidizerTM, a high-pressure homogenizer. Particle size, evaluated by ELS-Z, was about 64.5 nm. Results of this study revealed that signal transducer and activator of transcription 3 (STAT3) and NF-κB were indeed inhibited by L-AST, suggesting its anti-AD potential [20]. In fact, LIPs are characterized by a double-layered membrane, comparable to the phospholipidic cell membrane, surrounding an aqueous core, and are non-toxic and biodegradable delivery systems. Given their high biocompatibility, LIPs easily merge with the *stratum corneum* cells, allowing deep penetration into the epidermal layer [10]. When incorporating both hydrophobic and lyophobic drugs, they are also characterized by enhanced drug solubility, compatibility, and biodegradability, and they have been used to deliver drugs to specific affected sites [59].

### 5.2. β-Carotene

#### 5.2.1. Natural Source, physicochemical Features, and Bioactive Properties

β-carotene (Figure 1) belongs to the carotenoid family and is a vitamin A precursor, an important micronutrient for humans. It can be found in several natural sources, such as plants, marine algae, fungi, and bacteria [60]. Among carotene’s isomers (α, β, γ, δ, ε, and ζ), β-carotene is the most abundant and effective. β-carotene is known for its antioxidant activity and immune system stimulation. Its intake is documented as being useful in the prevention of allergic diseases, reducing the risk of AD [61,62]. Besides that, it also presents anti-inflammatory properties and is used in the treatment of several skin diseases, especially AD [21].

Structurally, β-carotene has a chemical backbone built by a polyene chain with a long conjugated double-bond system that ends with cyclic groups. There are no oxygen atoms in its composition, but its electron-rich conjugated system is responsible for its antioxidant property [60].

#### 5.2.2. Drug Delivery Systems and Pharmacological Activity

Kake and co-workers [62] have reported that β-carotene blocks inflammation by reducing inflammatory cytokine, factor and matrix metalloproteinase (MMP) activity in oxazolone-induced AD mice. Besides this, an increase in filaggrin’s expression was observed and it was concluded that, besides being a potent anti-inflammatory agent, β-carotene also improves the skin’s barrier function [62]. The same research group studied the oral effect of β-carotene on AD-like skin tissue and observed a significant suppression of TNF-α, IL-1β, monocyte chemoattractant protein-1 (MCP-1), thymic stromal lymphopoietin (TSLP), IL-6, IL-1β, IL-4, IL-5, and protease activated receptor 2 (Par-2). In addition, the expression of filaggrin was elevated. Moreover, β-carotene led to a reduced activity and/or mRNA expression of MMPs, degradation of the extracellular matrix, and regulation of chemokines [63].

Nanofibers (NFs) are nanomaterials that have several applications in the pharmaceutical field, given their properties such as large surface area–volume ratio [64]. Moreover, NFs are appreciated by the way that they reduce systemic absorption and the number of required drug administrations, besides the achievement of high production rates [65]. Among the polymeric NFs used for drug delivery systems, polycaprolactone (PLC) has been most frequently used given its good tissue compatibility and appropriate tensile strength. Semnani and co-workers [21] developed a PLC NF mat loaded with β-carotene. These mats were prepared by electrospinning, showing NFs 400–800 nm in diameter with desirable tensile properties. The in vitro degradability and drug release studies found a very slow degradability rate and gradual release of β-carotene. Results suggested the use of these β-carotene-loaded mats for the treatment of skin diseases such as AD.

### 5.3. Capsaicin

#### 5.3.1. Natural Source, Physicochemical Features, and Bioactive Properties

*Capsicum annum* L., a member of the Solanaceae botanical family, and widely known as chili pepper, is the main natural source of capsaicin (C_18_H_27_NO_3_) (Figure 1). This pungent and lipophilic alkaloid with a molecular weight of 305.40 g/mol represents more than 90% of all capsaicinoids present in chili pepper. Besides that, chili pepper is also the source of other capsaicinoid compounds such as dihydrocapsaicin, nordihydrocapsaicin, homodihydrocapsaicin, and homocapsaicin, all of them found in *Capsicum* fruits. The pungent property of capsaicinoids arises from the presence of an amide bond linking the acyl chain with the vanillyl ring. In fact, capsaicin has some similar structural features to piperine, another alkaloid. Such pungency has driven several bioactivities to be scientifically explored, such as the nociceptive, anti-inflammatory, anticarcinogenic, antiobesity, and antimicrobial activity [66,67,68].

#### 5.3.2. Drug Delivery Systems and Pharmacological Activity

The basis of analgesic activity of capsaicin is mainly related to the agonist activity upon the transient receptor potential vanilloid 1 (TRPV1) ion channel, which is expressed in nociceptive sensory nerves, namely C and some Aδ fibers, ultimately affecting the capacity of cutaneous sensory nerves to feel pain stimuli. On the other hand, its anti-inflammatory effect is evidenced by its suppressive action upon proinflammatory mediators such as COX-2 and iNOS. Despite the recognized nociceptive and anti-inflammatory properties of capsaicin, it has poor bioavailability due its lipophilic nature and potential skin irritation side effects, which have led some investigators to develop innovative nanosystems for the topical delivery of capsaicin [69].

Therefore, the report published by Cassano et al. (2022) [13] aimed at incorporating linolenic acid into SLNs based on curcumin, resveratrol, and capsaicin-derived esters for the treatment of AD. In this study, the results obtained for the curcumin and resveratrol monooleates, regarding the improvement of AD-like symptoms, were comparatively better than those obtained for the particles containing capsaicin oleate. These results are discussed further elsewhere in this paper. Nevertheless, the capsaicin SLNs presented an average size of 277.4 ± 12.0 nm, with a polydispersity index (PDI) of 0.192 *±* 0.095, and the entrapment efficiency (EE, %) was almost complete, reaching 99%. The advantages of SLNs include their highly accurate targeting to the affected sites on the skin, an improvement in the drug permeation into the dermal layer, controlled release, and decrease in systemic absorption, as well as avoidance of compounds’ degradation through hydrolysis and oxidation phenomena [13,22].

On the other hand, some authors have attempted an improved capsaicin topical delivery, disregarding possible pharmacological evidence directly related to AD, by only considering its anti-inflammatory and analgesic potential. From this perspective, Ghiasi et al. (2019) [69] developed an oil-in-water NE through the spontaneous emulsification methodology, aiming at creating an effective carrier for in vivo topical delivery of capsaicin, using Wistar albino rabbits. This NE was included in a cream and in a gel, and its safety and efficiency were compared to the conventional cream containing free capsaicin. According to skin irritation tests, there was no signs of ear edema or erythema, and rats’ paw edema decreased under treatment with these nanosystems, in comparison to the group treated with the conventional cream. Moreover, the analgesic activity of the capsaicin NE-based gel was evidenced, as rats better resisted the pain inflicted by a heat stimulus, plus it was revealed to be a better dosage form for the administration of the drug, improving skin permeability. NEs are isotropic binary systems, composed of two immiscible liquids, forming oil droplets with a particle size varying from 10 nm to 200 nm dispersed in an aqueous phase and stabilized by at least one surfactant [70]. The main advantage of NEs is the achievement of increased solubility of hydrophilic active ingredients, by dispersing them into the oily phase, thereby improving skin permeation [65]. This characteristic relies on the existence of positive charges that interact with negative charges of *stratum corneum* cells, enhancing percutaneous drug absorption [70].

For instance, Wang et al. [71] proposed capsaicin-loaded NLCs to increase skin permeation, encompassing the analgesic and anti-inflammatory potential of the molecule, while avoiding skin irritation. Similarly, Raza et al. [72] also applied NLCs for the topical delivery of capsaicin, improving the analgesic properties of this alkaloid and reducing skin irritation signs arising from its pungent property. NLCs are similar to SLNs, but consist of liquid content and a solid matrix instead, and have been suggested as cutting-edge lipid nanoparticles (NPs) for the treatment of AD. The application of NLCs presents fewer chances of drug leakage and increases drug loading, as well as drug half-life, controlled release, enhanced drug targeting, and entrapment efficiency [73].

### 5.4. Curcumin

#### 5.4.1. Natural Source, Physicochemical Features, and Bioactive Properties

The main sources of curcumin are the roots of *Curcuma longa* L., a plant widely known as turmeric that belongs to the same botanical family as ginger (*Zingiber officinale* L.), the Zingiberaceae family. Curcumin (C_21_H_20_O_6_) (Figure 1) is a β-diketone polyphenolic compound with unique structural characteristics, arising from the presence of β-diketo groups, carbon–carbon double bonds, and phenyl rings containing hydroxyl and methyl functional groups. Such structural features enable this potent antioxidant compound to target inflammatory cytokines, proteins, enzymes, as well transcription factors [8]. Curcumin, a bright yellow compound, has been traditionally used as a digestive facilitator, for gastrointestinal inflammation, and in skin ailments. Besides that, several in vitro and in vivo assays have attempted to validate its antimicrobial, anticancer, and anti-inflammatory properties [74]. Interestingly, it has been also employed to control AD symptoms in some Asian countries [8,74].

#### 5.4.2. Drug Delivery Systems and Pharmacological Activity

Curcumin has been proved to be an expression TSLP, through blockade of the caspase-1/NF-κB pathway, when tested in vitro on the human mast cell line HMC-1 [75]. Recently, mice were exposed to aerosolized ovalbumin (OVA), and the effect of curcumin in improving AD-induced symptoms was evaluated [8]. According to the results of this study, curcumin was shown to recover epidermal thickness and inhibit infiltration of inflammatory cells into the dermal layer. At the molecular level, it was observed that, under curcumin treatment, the Th2-promoting cytokines (TSLP/IL-33) and Th2 cytokines (IL-4/IL-5/IL-13/IL-31) have their expression inhibited, as well as STAT-6 phosphorylation and GATA-3 expression [8].

Therefore, Zhu et al. [23] and colleagues have designed novel curcumin-loaded zein–silk sericin NPs, for the delivery of this polyphenolic compound and to enhance its skin penetration into the dermal layer, thereby aiming at reducing AD symptoms, besides presenting minimal side effects. Briefly, in this study, NPs were prepared by injecting zein hydroalcoholic solutions into silk sericin protein dispersions, followed by curcumin encapsulation through a facile antisolvent route. Particles varied from 330 to 400 nm in size, showing a zeta potential (ZP) of −22 to −25 mV, and the PDI varied from 0.29 to 0.49. The formulated nanocarriers (zein-to-silk sericin mass ratios of 1:0.25) showed the best penetrating behavior (240 µm in depth) into porcine cortex, including cuticle, epidermis, and dermis, which shows the efficiency of the formulated transdermal delivery system. Moreover, the designed NPs suppressed inflammatory cytokines and chemokines, through the inhibition of the nuclear translocation of NF-κBp65, in comparison to free curcumin, when tested in an in vitro AD cell model (HaCaT cell line).

In another study, gels containing SLNs were loaded with tetrahydrocurcumin, a curcumin-derived metabolite with certain pharmacological therapeutic advantages, besides presenting a greater polarity, over curcumin itself [22]. The nano-based system was obtained through a modified microemulsion technique, followed by a high-speed homogenization approach that ended with an increased tetrahydrocurcumin loading. Following this methodology, a high drug EE of 83.10% *±* 2.29% was achieved, and the particles were 109.2 nm in size. Afterwards, the SLN dispersion was included in a Carbopol (2% *w*/*v*) hydrogel. In the in vivo assays, using 2,4-dinitrochlorobenzene (DNCB)-induced AD mice, the anti-inflammatory potential of this nanosystem was evidenced, as it decreased expression levels of TNF-α and IL-6, and following the histopathological analyses, complete healing of AD-like lesions was observed. More importantly, AD-like symptom alleviation was significantly different (*p ≤* 0.05) from that produced by the marketed ointment Tacroz^®^ Forte, or even the produced gel bearing free tetrahydrocurcumin. Furthermore, the tetrahydrocurcumin-bearing NPs also ameliorated skin hydration, as shown by a great transdermal penetration through the skin layers into the dermis [22].

Another recent study realized the encapsulation of linolenic acid into SLNs that were able to penetrate deeply into the skin. These SLNs were loaded with curcumin, as well as other natural molecules, such as resveratrol and capsaicin [13]. Firstly, esterification reactions with oleic acid were carried out to produce curcumin and resveratrol monooleate and capsaicin oleate, followed by a microemulsion methodology to prepare SLNs. The curcumin monooleate presented an EE of 62%, compared to 85% and 99% for the capsaicin- and resveratrol-produced esters, respectively. Focusing on the obtained results for the curcumin-based formulations, they were 493.6 *±* 183.90 nm in size and showed a PDI of 263 *±* 0.043, which indicates homogeneity in the distribution of the particle size. Furthermore, these systems were not cytotoxic when tested on NCTC 2544 and THP-1 monocytes differentiated into M2 macrophages, even increasing cell viability in some cases, which was also observed for the resveratrol SLNs. Regarding the anti-inflammatory potential, the authors observed that the curcumin SLNs significantly suppressed the production of IL-6, both in basal conditions and in the presence of TNF-α, used as a proinflammatory stimulus.

The inclusion of curcumin into SLN-containing gels has also been attempted but regarding the treatment of irritant contact dermatitis and skin pigmentation [76]. On the other hand, a work has been carried out to overcome some curcumin delivery drawbacks and it provided insights that the formulated LIPs may serve as vehicles for a broad dermatological application, including AD [77]. Therefore, neutral, cationic, and anionic deformable LIPs were formulated. According to the main findings, the cationic deformable LIPs presented the most appreciable properties, namely, they enhanced penetration of curcumin through the full thickness of human skin and they provided the most interesting retention of the compound. Moreover, these LIPs showed potent in vitro anti-inflammatory activity, besides the absence of cytotoxicity in human skin fibroblasts, along with evidence of cell proliferation stimulation [77].

### 5.5. Cynaroside

#### 5.5.1. Natural Source, Physicochemical Features, and Bioactive Properties

Cynaroside (luteolin-7-O-glucoside or luteoloside; C_21_H_20_O_11_) (Figure 1) is a natural product found in *Bidens tripartita* L., *Verbascum lychnitis* L., *Elsholtiza bodinieri* Vaniot, and other plants. This glycosyloxyflavone is functionally related to luteolin [24,78].

It is known by its diaphoretic, diuretic, antiseptic, anti-inflammatory and antiallergic activities [24]. Anticancer effects [79] and activity against hepatitis B [80] are also known.

#### 5.5.2. Drug Delivery Systems and Pharmacological Activity

Cynaroside exerts its anti-inflammatory effect by inhibiting the expression of IL-4 and IgE [70]. It also blocks IL-22 and the IL-6/STAT3 pathway which contributes to control of keratinocyte hyperproliferation [24,81]. The anti-inflammatory effect was also evaluated in vitro, revealing a decrease in the production of NO and ROS generation. In vivo anti-inflammatory evaluation was performed using a xylene-induced auricular swelling mouse model and it showed inhibition of edema and a decrease in prostaglandin E2 (PGE_2_) of mice [78].

Szekalska and co-workers [24] prepared novel hydrogels as topical carriers for cynaroside. They used the anionic polymer alginate for its bioadhesive properties. Alginate was mixed with glycerol and propylene glycol, followed by the inclusion of crushed cynaroside that had been obtained from aerial parts of *B. tripartita*. Particle size ranged from 22,000 to 26,000 nm. The in vivo anti-inflammatory and antiallergic activities were measured using skin from hairless mice. For the anti-inflammatory activity, the carrageenan-induced mouse paw edema test was used, and for the antiallergic activity, the oxazolone-induced ear inflammation model. Results revealed that 5% and 10% cynaroside hydrogels substantially reduced skin and tissue inflammation, and inflammatory infiltrates. Hence, the topical application of cynaroside allows the reduction of the number of T and mast cells and histiocytes in mouse skin with inflammation and AD, which supports the idea that flavonoids, like cynaroside, can hinder the overexpression of cytokines and IgE levels [82].

To overcome cynaroside’s poor solubility, bioavailability, and oral absorption, Qing et al. [83] prepared biodegradable and biocompatible diblock copolymer micelles loaded with cymaroside, creating water-soluble co-polymer micelles. These micelles have a hydrophobic core, where the active substance is placed, and a hydrophilic shell. Encapsulation was carried out using methoxy polyethylene glycol-polycaprolactone (mPEG-PCL), methoxy polyethylene glycol-polylactide-co-glycolide (mPEG-PLGA), and methoxy polyethylene glycol-polylactide (mPEG-PDLLA). The self-assembly method created water-soluble torispherical micelles with an average diameter of 70 nm. The mPEG-PLGA showed the higher loading capacity, while mPEG-PCL had better stability. In vitro drug release showed a 30% cynaroside release from micelles [83]. Before making these micelles, Qing and co-workers [84] investigated a nanocomposite material made of nanocrystalline cellulose (NCC) to improve cynaroside’s bioavailability. NCC has been used in biomedical fields in drug delivery systems because of its biocompatibility, biodegradability, and low cytotoxicity. These last two systems mentioned are valuable formulations, but they were not tested for AD or another inflammatory-based skin conditions.

### 5.6. Dictamnine

#### 5.6.1. Natural Source, Physicochemical Features, and Bioactive Properties

*Dictamnus dasycarpus* Turcz. is a traditional Chinese medicinal herb frequently used in China, Japan, and Korea to treat inflammatory-related skin diseases such as AD, pruritus, and urticaria [30].

From the root bark of *D. dasycarpus,* dictamnine (C_12_H_9_NO_2_) (Figure 1) was extracted, the main compound of which has been revealed to possess several bioactivities, such as anti-inflammation, antiangiogenic, anticancer, antifungal, antibacterial and antiyeast [25]. Dictamnine is a furoquinoline alkaloid [85].

#### 5.6.2. Drug Delivery Systems and Pharmacological Activity

Dictamnine’s anti-inflammatory mechanism has not been precisely described [25]. However, there are several findings concerning *D. dasycarpus* extract’s anti-inflammatory effects. Chang and colleagues [86] showed that it protected skin cells from oxidation and inflammation by attenuating ROS, TNF-α, IL-1, and IL-6 levels, and by modulating antioxidant enzyme activity, cell signaling pathways, and the expression of NF-κB in keratinocytes. Their results suggested it to be interesting in preventing the inflammatory mechanism in dermatitis. Yang et al. [87] studied the extract´s effect in contact dermatitis mice. These were sensitized by application of DNFB in acetone/olive oil onto the ears’ dorsum and the results showed that it inhibited the production of TNF-α, IFN-γ, and IL-6. These effects led to ameliorated skin lesions by reducing epidermal hyperplasia, hyperkeratosis, and spongiotic changes.

Recently, Yang et al. [88] have studied the anti-inflammatory and antipruritic effects of dictamnine in an AD mouse model. Results showed an efficient inhibition of AD-induced chronic itch, epidermal thickness, inflammation, and inflammatory cell infiltration. A decrease in the expression of Mas-related G-protein-coupled receptor A3 (MrgprA3) and transient receptor potential channel A1 (TRPA1), the signaling pathways used for the development of chronic itch, was also observed. These data are consistent with dictamnine being interesting for the treatment of chronic itch associated with AD.

Focused on studying dictamnine’s efficacy and mechanism as an anti-inflammatory in AD, Lin’s group [25] developed a nanoformulation, PLGA-nanocarrier-encapsulated dictamnine (Dic-PLGA-NC). The nanoformulated dictamnine had a particle size of nearly 186 nm and a PDI of 0.146. As for the encapsulation efficiency and loading capacity, high-performance liquid chromatography (HPLC) results showed them to be 93.7% and 51.8%, respectively. In the mouse model created for studying AD, results showed that these nanocarriers were able to penetrate 300 μm deep, reaching dermal tissue and allowing a sustained release of dictamnine from PLGA carriers. As for anti-inflammation effects using the new formula, results showed a reduced TSLP, IL-1β, and TNF-α expression, and an apparent improvement of skin inflammation was observed in treated mice, whose allergic dermatitis was induced via oxazolone [25].

### 5.7. Epigallocatechin-3-gallate

#### 5.7.1. Natural Source, Physicochemical Features, and Bioactive Properties

Epigallocatechin-3-gallate (EGCG) (Figure 1) is a polyphenol, part of the catechin subclass, and is mostly found in the leaves of green tea, *Camellia sinensis* (L.) Kuntze (Theaceae family). Focusing on green tea catechins, EGCG represents more than 50% of those compounds, and about 16.5% of the water-extractable fraction of tea. A cup of tea may contain about 200–300 mg of EGCG. Despite the relevance of EGCG, there are other important catechins in tea, such as (-)-epicatechin (EC), (-)-epicatechin-3-gallate (ECG), and (-)-epigallocatechin (EGC), all of which differ with respect to their pharmacodynamics and pharmacokinetic properties, which are intimately related to structural features. For example, it is considered that the existence of hydroxyl groups at the 3′, 4′, and 5′ C positions on the B ring of the EGCG molecule and the galloyl moiety esterified at carbon 3 on the C ring are key points explaining the great antioxidant activity. Besides that, other properties have been attributed to EGCG, such as anticancer, vasoprotective, and anti-inflammatory activities [89].

#### 5.7.2. Drug Delivery Systems and Pharmacological Activity

EGCG and other tea catechins have been highlighted for their beneficial effects on skin-related conditions. From this perspective, Noh et al. [90] were pioneers in investigating the anti-inflammatory role of EGCG when topically applied on the skin of an AD mouse model (NC/Nga) induced by 1% *Dermatophagoides pteronissinus* extract. The findings suggest that total clinical severity score and ear swelling were significantly reduced (*p* < 0.05) after EGCG treatment, along with a histopathological grading improvement. Noteworthily, the mRNA expression of the cytokines macrophage migration inhibitory factor (MIF), TNF-α, interferon gamma (IFN-γ), IL-2, and IL-12 p40 was significantly diminished by EGCG (*p* < 0.05) in the AD skin lesions, which was also observed in the immunohistochemistry assays. Moreover, the elevated serum MIF and IgE levels also suffered a significant reduction (*p* < 0.05). Altogether, these findings suggest that EGCG suppresses MIF and T helper 1 cytokines, thus leading to an improvement in AD skin lesions induced by DPE [90].

Since catechins such as EGCG have been highlighted for their outstanding pharmacological activity on the skin, including the wound-healing effect, antiaging, antiacne, antipsoriatic properties, and, more importantly, the effect on AD, several strategies have been attempted for their nanoencapsulation [91]. The work of Drew et al. [14] showed that gelatin/EGCG nanoparticles (GE NPs) were efficient in reducing IL-6 and IL-8 inflammatory factors, using an in vitro model of lipopolysaccharide (LPS)-inflamed WS1 dermal fibroblasts, at non-toxic concentrations lower than 10 µg/mL. Furthermore, in vivo assays conducted on nude mouse skin (BALB/cAnN.Cg-Foxn1nu/CrlNarl mice) also showed that GE NPs present skin absorbance but do not cause adverse effects. In this study, the formulated GE NPs were prepared following a self-assembly mechanism, and NPs showed an average size of 112.5 ± 19.09 nm, a positive ZP 23.2 ± 0.5 mV, and a PDI of 0.3 ± 0.05 [14].

Recently, Han et al. [15] have created polyethylene glycol–PLGA–EGCG nanoparticles (EGCG-NPs) following the double emulsion methodology. The produced formulation presented an average size of 176.2 nm, the zeta potential was −33.3 mV, and the entrapment efficiency was 86% while PDI was 0.044. In addition to these data, EGCG-NPs were shown to be spherical in shape, did not suffer aggregation or adhesion, and presented regular arrangement. Additionally, EGCG-NPs provided a significant improvement of AD symptoms and skin lesions, namely a diminishment of skin and ear thickness, dermatitis score, and scratching behavior, when using an AD in vivo model of Kunming mice treated with DNCB. In addition, the EGCG-NPs led to an improvement in AD-related oxidative stress, by elevating the activities of antioxidative enzymes such as superoxide dismutase (SOD) and glutathione (GSH), even prior to the end of the study. Noteworthily, the expression levels of inflammatory cytokines such as Th1 (IFN-g and TNF-α), Th2 (IL-4 and IL-6), and Th17 (IL-17A) were significantly down-regulated when compared to the control group following a time-specific pattern. Consequently, receptor-interacting protein 1 (RIP1), receptor-interacting protein 3 (RIP3), and mixed lineage kinase domain-like pseudokinase (MLKL) proteins also had their overexpression blocked upon topical treatment with EGCG-containing particles, demonstrating that necroptosis is inhibited instead of apoptosis. Similarly, the expression of phosphorylated p38 (p-p38), extracellular signal-regulated kinase 1 (ERK1), and extracellular signal-regulated kinase 2 (ERK2) was blocked as well. Finally, the authors also showed that alleviation of AD symptoms was due to MAPK blockage. This drug delivery system is a promising strategy in AD therapeutics as it improved the redox status, preserved the balance between Th1 and Th2 inflammatory factors, and targeted necroptosis instead of apoptosis in DNCB-treated mice [15].

Similarly, epigallocatechin gallate/L-ascorbic acid-loaded poly-*γ*-glutamate microneedles also proved to be a successful approach to alleviate AD-related symptoms, when administered once a week, by topically applying it on the skin of a DNCB-treated mouse model. This report shows that this drug delivery system was successful in reducing dermatitis score along with inhibition of mast cell infiltration, plus reduction of the expression of IFN-*γ*, Th2 cytokine secretion, IgE, and histamine [31].

### 5.8. Glycyrrhizic Acid

#### 5.8.1. Natural Source, Physicochemical Features, and Bioactive Properties

The roots of *Glycyrrhiza glabra* L. (Fabaceae), also commonly known as liquorice, are the source of glycyrrhizic acid (GA) (Figure 1), that confers to the roots a typical sickly sweet taste. Also known as glycyrrhizin (C_42_H_62_O_16_), GA is a pentacyclic triterpenoid saponin glycoside, with a molecular weight of 822.92 g/mol, that can be found in the form of two stereoisomers, 18α-glycyrrhetinic acid and 18β-glycyrrhetinic acid, both formed after hydrolytic reactions promoted by intestinal bacteria or in situ by the action of the plant’s glucuronidase enzyme. The hydrophilic part of the molecule is represented by glucuronic acid, while glycyrrhetic acid residue corresponds to the non-polar part. Besides these saponin-like compounds, flavonoids and polysaccharides are other important bioactive molecules. It has been mentioned that GA acts as an antiviral, anti-inflammatory, anticancer, antimicrobial, antidiabetic, and hepatoprotective compound. In fact, most of the pharmacological activity of liquorice arises from GA alone [92,93].

The root’s extract and GA are well known for their beneficial effects as antioxidants and anti-inflammatories in topical applications. Such effects are assumed to positively influence contact and atopic dermatitis and other inflammatory skin ailments, such as sunburns or acne vulgaris. Most of these diseases present inflammatory signs such as pruritus, erythema, or even skin pigmentation [93].

#### 5.8.2. Drug Delivery Systems and Pharmacological Activity

Using a DNCB-treated mouse model of AD, investigators have shown that GA mainly acts by inhibiting the high-mobility group box1 (HMGB1) signaling pathway. In addition, this natural molecule also suppressed the expression of the receptor for advanced glycation end products (RAGEs), the phosphorylation of NF-κB, and the infiltration of mast cells. Given the recognized anti-inflammatory value of *G. glabra* and major compounds such as GA, 18beta-glycyrrhetinic acid, isoliquiritin, and liquiritigenin, these have also been tested concerning their inhibitory effects on inflammatory and allergic reactions such as AD [93,94].

The thin film hydration method was employed to produce transfersomes (TRAs) loaded with GA, that were further included in a hydrogel, as a vehicle for the GA–transfersomal suspension [16]. The GA-loaded TRAs presented a particle size varying between 270.40 and 56.94 nm, PDI ranged between 1.00 and 0.13, and the ZP was −4.76 mv. The GA–trans-loaded hydrogel presented a ZP of 36.4 mv. Moreover, the EE was shown to be improved by an increasing content of the lipidic fraction of TRAs, thus resulting in an EE ranging between 66.23 ± 0.61% and 93.10 ± 0.3%. According to the in vitro drug release study, a drug cumulative pattern was evidenced, reaching a drug release percentage of 89.8% at up to 24 h. Meanwhile, ex vivo permeation was only 5.8% at up to 24 h, thus indicating that the drug effectively is deposited on the skin. Such deposition is required for the management of AD, since the drug should not permeate into the skin and suffer systemic absorption, thereby exerting its topical therapeutic effect. Moreover, in comparison to other groups, GA–trans-loaded gel led to a significant reduction in erythema signs and scratching behavior in the in vivo assays, using DNCB-induced AD in BALB/c mice [16]. For instance, ammonium glycyrrhizate, which is a derivative salt of GA, was also included in TRAs, realizing an improvement in topical administration of this anti-inflammatory compound [95]. TRAs are innovative ultra-deformable vesicles, consisting of a single-chain surfactant which is the edge activator, a lipidic part, and solvent. These nano-based technology are similar to LIPs and ethosomes (ETOs). TRAs have edge activators that give them the capacity to become ultra-deformable and highly elastic, squeezing themselves and penetrating across *stratum corneum*, resulting in a higher permeation ability [7,16].

18β-glycyrrhetinic acid nanocrystals were prepared by a high-pressure homogenization method. Afterwards, GA nanocrystalline suspension presented an average size of 288.6 ± 7.3 nm and a PDI around 0.13 ± 0.10, while the thermal stability and crystallinity decreased, but solubility increased significantly after nanocrystallization. In comparison to coarse GA hydrogel and the positive control group represented by the drug indomethacin, the formulated nano-GA hydrogel provided better anti-inflammatory activity, by decreasing the signs of ear edema and levels of proinflammatory cytokines, reduced myeloperoxidase activity, as well as reduced infiltration and aggregation of neutrophils. Although these results are not specifically in the AD context, the authors suggest that these nanocrystals may be useful in the treatment of skin diseases in general [96]. In another study, a modified LIP-like vesicle loaded with GA had some changes in core ingredients, by including ethanol and glycerol, aiming at an improvement of the stability of the nanosystems and to promote efficacious penetration of the drug into the skin. This modified formulation, called glycethosome, was prepared by an ethanol injection and sonication technique and showed a mean particle size of 94.5 nm, a PDI of 0.216, and 99.8% EE, when the formulations contained glycerol at 50% and ethanol at 25%. Moreover, at the these concentrations, glycethosomes showed the smallest particle size and the best stability, besides improving the transdermal effect [97].

### 5.9. Guar Gum

#### 5.9.1. General Considerations

*Cyamopsis tetragonoloba* (L.) Taub. is a leguminous plant and from the endosperm of its bean seeds is extracted the so-called guar gum (Figure 1) [98]. This is a water-soluble, non-ionic polysaccharide with a molecular weight of 535.14 g/mol and a viscous to gel-like consistency [99]. Chemically classified as a galactomannan, it contains a straight chain of D-mannose units linked by β(1-4) glycoside linkages and a single D-galactose unit (2:1 ratio) [98]. Despite its main current use in cosmetic and food industries as a stabilizing agent and in the pharmaceutical industry in drug microencapsulation, it has also been used for its medicinal properties. Guar gum is effective in lowering postprandial glucose and cholesterol, and there are also reports of its antimicrobial and antiproliferative activity [26,98].

#### 5.9.2. Drug Delivery Systems and Pharmacological Activity

Ghosh and co-workers prepared guar gum NPs (GNs) and explored their therapeutical effect in AD, in vitro and in vivo [26]. GNs were prepared by acid hydrolysis from guar gum dispersed in water, without any surfactant, affording spherical NPs with a size range of 30–80 nm [27]. The in vitro study showed the successful wound-healing effect of GNs and the in vivo test, performed on topically oxazolone-sensitized mice, also revealed a successful decrease in AD symptoms, such as redness and epidermal thickness. A decrease in serum IgE levels and total counts for blood cells, skin cells, eosinophils, macrophages, and neutrophils was also registered. They concluded that GNs are useful agents as anti-inflammatory, antiallergic, and proregenerative agents, being efficient in ameliorating AD [26].

### 5.10. Hederagenin

#### 5.10.1. Natural Source, Physicochemical Features, and Bioactive Properties

Hederagenin (Figure 1) is a pentacyclic oleane-type triterpenoid acid found in the pericarp fruits of *Sapindus saponaria* L. (Sapindaceae) and the buds of *Lonicera japonica* Thunb. These have been traditionally used for the treatment of skin conditions and the dried buds have also revealed anti-AD effects [100,101]. Hederagenin has two hydroxyl groups in the A ring, a double bond in the C ring, and a carboxylic group at C-28. There are several reports about the different biological properties of this natural compound, such as anti-inflammatory, antimicrobial, and anticancer [102].

#### 5.10.2. Drug Delivery Systems and Pharmacological Activity

Hederagenin has an anti-inflammatory effect by regulation of MLK3 signaling, attenuation of the inflammatory cytokines TNF-α, IL-1, and IL-6, and by decreasing other proinflammatory factors such as TNF-α and COX-2 [102,103].

In this sense, hederagenin was used to coat maghemite (γ-Fe_2_O_3_) nanoparticles (HMs) and studied for its immunomodulatory and anti-inflammatory efficacy in AD [28]. Results revealed a dose-dependent inhibition of AD-related cytokines, including IFN-γ, TNF-α, IL-4, IL-6, IL-17, and TSLP. In vivo studies, conducted in mice with AD-like lesions created in their ear skin using repeated *Dermatophagoides farinae* extract and DNCB, also showed a reduction in mast cells’ infiltration, lowered epidermal and dermal thickness of skin, and relieved lumping lymph nodes. These results reveal the HM synergistic effect (hederagenin and maghemite) with anti-inflammatory and immunomodulatory activities, hence having great potential for AD medication. The HMs were prepared using the emulsion method, by mixing the maghemite NPs that were first prepared with a solution of hederagenin. The obtained HMs were round NPs with an average size of 10.9 nm [28].

### 5.11. Piperine

#### 5.11.1. Natural Source, Physicochemical Features, and Bioactive Properties

Considered to be the major alkaloid (approximately 98%) found in black pepper (*Piper nigrum* L.), piperine (C_17_H_19_NO_3_) (Figure 1) is an alkaloid mainly found in the oleoresins of plants from the genus *Piper* (Piperaceae family), with amounts ranging from 2 to 9%, depending on the plant species used for extraction. The socioeconomic value of peppers, in general, is due to the flavor and pungency arising from piperine, but also from essential oils found in peppers’ oleoresins. As for most alkaloids, piperine is a poorly water-soluble compound and has a very weak basis, easily solubilizing itself in the presence of acids or alkalis. According to ancient Chinese and Indian medicine practices, black pepper was used for pain relief, rheumatism, and fever and as a circulatory, digestive, and appetite stimulant. more recently, piperine has been studied for its antioxidant, chemopreventive, and anticancer pharmacological activities, among others [104].

#### 5.11.2. Drug Delivery Systems and Pharmacological Activity

Interestingly, the immunomodulatory and anti-inflammatory potential of this alkaloid was explored by testing black pepper fruit extract in allergic contact dermatitis. The oral administration of piperine to mice showed an inhibitory effect upon eosinophils, IgE, and especially Th2 cytokine expression, which points to the potential of piperine in other inflammatory skin ailments [105]. In another work carried out by the same team, using a trimellitic anhydride (TMA)-induced AD-like mouse model, it was demonstrated that topical application of piperine resulted in the suppression of immune responses regulated by Th2 cytokines, noteworthily including the STAT6/GATA3/IL-4 signaling pathway [106].

From this perspective, one outstanding study was developed attempting the topical administration of piperine by including it in ETOs, thus overcoming the solubility and delivery issues, while exploring it as a therapeutic agent for AD [32]. ETOs are phospholipid-based flexible and elastic vesicles bearing an ethanolic core (20–45% ethanol), but also containing other key ingredients such as phosphatidylcholine, cholesterol, and water. Given the high content of ethanol in these nanocarriers, they have the capacity to easily penetrate the epidermic *stratum corneum*, thus promoting a deep and localized drug delivery into the skin [7,10,32,65]. According to the study of Kumar et al. [32], piperine-loaded ETOs were prepared by the cold method, and for optimized ethosomal dispersion, the nanocarriers presented an EE of 74.30 ± 3.88% and a vesicle size of 318.1 nm. In addition, the ZP of the formulated vesicles was 32.6 mV, and they were spherical in shape. Regarding the in vitro cytotoxicity assays, the creams were non-toxic when tested in HaCaT cell lines. In ex vivo assays, the fabricated ETO-based creams easily penetrated the skin, mainly being deposited at the epidermal and dermal layers. In comparison to the negative control, the ethosomal and conventional creams containing piperine at 0.1% and 0.125%, respectively, both significantly reduced the ear and skin thickness, skin severity, white blood cells, granulocytes, and IgE antibody levels in the BALB/c mouse model. Finally, given the efficiency of the piperine ethosomal cream in reducing in AD markers, comparing to tacrolimus (0.1%) and conventional cream applications, the authors suggested that this formulation has great potential for the management of mild to moderate AD [32].

Nevertheless, regarding the generality of skin inflammation diseases, a very recent investigation was reported on the development of piperine-loaded NPs included in hyaluronic acid/sodium alginate-based membranes [107]. The nanoprecipitation technique was used to produce the polymeric NPs composed of Eudragit S100 and Poloxamer 188, resulting in spherical NPs with a mean diameter size of 122.1 ± 2.0 nm, a PDI of 0.266, and an EE of 76.2%. Afterwards, hyaluronic acid/sodium alginate membranes were produced for the subsequent incorporation of the synthesized NPs. The main results suggest that the produced formulation evidenced a reduction of the mouse ear inflammatory symptoms of nearly 46%, besides the absence of cytotoxic adverse effects on the L929 mouse fibroblast cell line [107].

### 5.12. Quercetin

#### 5.12.1. Natural Source, Physicochemical Features, and Bioactive Properties

Quercetin (Figure 1) (C_15_H_10_O_7_) is a widespread flavonol found in several daily food products, such as fruits (berries, grapes, nuts, and apples), vegetables (onions, tomatoes, and cabbages), and beverages such as tea and red wine, besides its presence in well-recognized medicinal plants such as *Sambucus nigra* L., *Hypericum perforatum* L., and *Ginkgo biloba* L. [108]. Quercetin is a water-insoluble molecule, while easily solubilizing itself in alcohol, acetic acid, and lipids. In nature, quercetin is often found bonded to other molecules that may enhance the solubility of the aglycone, namely sugars, forming quercetin glycosides, such as quercetin-3-*O*-glucoside, an important pigment in vegetables and fruits [2,109]. Structurally, quercetin bears four active groups, namely a dihydroxy group between the A ring, *O*-dihydroxy group B, C ring C2, C3 double bond, and 4-carbonyl [2,108]. In addition, the presence of several OH groups and double bonds confers to this flavonoid a strong antioxidant activity [108,109]. Besides that, several other beneficial skin-related effects have been reported, namely wound healing, antipsoriatic, photoprotective, anti-inflammatory, and skin whitening, thus justifying the critical role of this molecule in cosmetics and pharmaceuticals acting on the skin [109].

#### 5.12.2. Drug Delivery Systems and Pharmacological Activity

Although numerous studies have been carried out exploring the molecular effects of quercetin, either in vitro or in vivo, as well as some clinical trials, the truth is that the exact antioxidant, antiallergic, and anti-inflammatory mechanisms are not fully uncovered [110]. As an example, the anti-inflammatory mechanisms of quercetin, along with those of the flavonol galangin, were assessed in vitro in LPS-stimulated RAW264.7 macrophages, and in vivo by using DNCB-treated mouse models of AD. In this investigation, the authors found that NF-κB, ERK1 and 2, and c-Jun N-terminal kinase (JNK) may be potential molecular targets of quercetin, as well as of galangin. In addition to these findings, oral administration of both flavanols to DNCB-treated mouse models of AD also led to a decrease in inflammation, as the compounds decreased ear edema as well the levels of serum IgE [111].

An in vitro model of AD was used, by stimulating HaCaT keratinocytes with proinflammatory factors such as IL-4, IL-13, and TNF-α, to induce an in vitro AD model. The anti-inflammatory and antioxidant power of quercetin in the AD context was unveiled, when cells’ pretreatment with quercetin (1.5 µM) led to a decrease in the expression of IL-1b, IL-6, IL-8, and TSLP and an improvement of the oxidative cellular defenses by an augmentation of the expression of SOD1, SOD2, catalase, glutathione peroxidase, and IL-10. On the other hand, quercetin also evidenced its wound-healing potential mainly by the targeted inhibition of MMP1, MMP2, and MMP9 and by a decrease in phosphorylation of ERK1 and 2 in the MAPK pathway, as well the expression of NF-κB, while the phosphorylation of STAT6 remained unaltered [112].

In this regard, some efforts to enhance the delivery and bioavailability of the molecule have been carried out. Therefore, the method of emulsion evaporation–solidification at low temperature was employed to develop quercetin-loaded NLCs [12]. The characterization of these formulations revealed that particles were spherical in shape and presented a particle size of 215.2 nm, ZP was −20.10 ± 1.22 mV, mean EE was 89.95 ± 0.16%, while drug loading was 3.05%. According to the results, in comparison to a quercetin propylene glycol solution, the developed nanosystem increased the amount of drug retention in epidermal and dermal skin layers, while revealing an easy percutaneous permeation across the *stratum corneum*. On the other hand, the in vivo assays using male Kunming mice evidenced that these NLCs also improved inflammation symptoms and had an enhanced antioxidant effect, thus proving them to be an efficient topical delivery system for AD management [12].

### 5.13. Resveratrol

#### 5.13.1. Natural Source, Physicochemical Features, and Bioactive Properties

Resveratrol (Figure 1) is a stilbene polyphenol, also considered to be a phytoalexin, as it is involved in plant defense against abiotic and biotic hazards, such as UV radiation and fungal infections, respectively, which in turn usually leads to an increase in its synthesis in plant tissues [113,114]. Resveratrol (C_14_H_12_O_3_) has a molecular weight of 228.25 g/mol and a melting point of 254 °C, easily dissolving in alcohols such as ethanol and acetone, but poorly dissolving in water [114]. Among plants, UV radiation-mediated reactions may lead to the isomerization of the bioactive form *trans-*resveratrol to the *cis* isomer, both found in plants’ tissues [113,114]. Although resveratrol was first identified in the roots of white hellebore (*Veratrum grandiflorum* O. Loes), vines and red grapes’ skin (*Vitis vinifera* L., Vitaceae) are by far the major sources of resveratrol [114,115,116]. In addition, this stilbenoid is also found in several berries such as blueberries and cranberries, peanuts, cocoa, and tomatoes. Although some adverse effects have been reported for resveratrol, its bioactive properties are outstanding, namely the anti-inflammatory, antimicrobial, anticancer, antiaging, cardioprotective, vasorelaxant, phytoestrogenic, and neuroprotective activities, besides being a well-validated antioxidant protector, given its action as a strong radical scavenger. Specifically, the antioxidant power of resveratrol arises from two phenolic rings connected by a double bond [2,116]. The antiaging and antioxidant properties have allowed the pure compound to be included in cosmetics at concentrations rising to 5%, or even in the form of extract or derivative-like compounds [115]. Despite its natural sources, both chemical and biological synthetic approaches have been employed for large-scale obtention of the compound, namely through *Saccharomyces cerevisiae* fermentation [116].

#### 5.13.2. Drug Delivery Systems and Pharmacological Activity

The potentiality of resveratrol in AD management was investigated in DNCB-induced NC/Nga mice and in an in vitro 3D skin model [117]. In this study, resveratrol-enriched rice obtained through genetic engineering was included. Rice (*Oryza sativa* L. var. *japonica*) was considered given its many recognized skin-associated benefits, and therefore the synergistic effect with resveratrol was investigated by combining both natural products. The investigation was carried out over five weeks, and it was found that the resveratrol-enriched rice markedly suppressed dermatitis score, scratching behavior, and transepidermal water loss. Moreover, serum IL-31 and IgE levels, as well the production of IL-6 in keratinocytes, were suppressed following resveratrol-enriched rice treatment [117]. In addition, the work carried out by Karuppagounder et al. [118], evidenced that oral administration of resveratrol (20 mg/kg/day) in NC/Nga mice attenuates DPE-induced AD-like symptoms, by causing the suppression of several inflammatory patterns such as HMGB1, RAGE, Toll-like receptor (TLR)4, NF-κB, phosphatidylinositide 3-kinase (PI3K), ERK1 and 2, COX-2, TNF-α, IL-1β, and IL-2Rα [118]. Finally, this scientific report suggested that resveratrol potentially targets AD disease by modulating protein expression in the HMGB1 pathway. On the other hand, resveratrol treatment (30 mg/kg/day) for 6 weeks showed that the stilbene was effective against AD-like inflammation symptoms in BALB/c mice, by targeting epithelial apoptosis through caspase-3 and epithelium-derived cytokines such as IL-25, IL-33, and TSLP. In this work, an improvement in epithelial thickness was also observed [119].

Recently, the encapsulation of linolenic acid into SLNs with resveratrol, curcumin, and capsaicin has also been attempted [13]. In this study, esterification reactions led to the obtention of resveratrol monooleate, as well as esters for the other bioactive molecules. The EE of resveratrol was 85%, comparatively higher than that obtained for curcumin (62%). The SLN resveratrol-based formulations were 271.8 ± 4.0 nm in size and showed a PDI of 0.005. The resveratrol nanosystems presented no cytotoxic effects when tested on NCTC 2544 and THP-1 monocytes differentiated into M2 macrophages. In comparison to the SLN without linolenic acid, the ones that contained resveratrol markedly suppressed the production of MCP-1, a key cytokine for the recruitment of monocytes under an inflammation scenario, and also decreased the production of IL-6 under TNF-α stimulus. Besides that, the resveratrol-containing NPs presented the best antioxidant activity in comparison to the other formulations [13].

The rising interest in resveratrol potentialities has led investigators to incorporate it into different nanosystems for multiple skin applications. For example, SLNs containing the seed butter of *Theobroma grandiflorum* (Willd. ex Spreng.) K. Schum. as the lipidic core were designed for the controlled topical delivery of resveratrol as the active principle. This formulation presented antioxidant potential, as well permeation and drug accumulation in the upper skin [120]. Meanwhile, Sun et al. [121] and colleagues performed a comparative study, by preparing NEs, SLNs, and NLCs through the hot high-pressure homogenization technique. The authors demonstrated that the lipidic ratio and composition of the lipid-based formulations highly influence the resveratrol delivery and retention on the skin. Moreover, evidence was gathered showing that a high lipid ratio of the formulation may improve resveratrol topical release [121]. It is worth mentioning that resveratrol SLNs incorporated into a Carbopol gel have also been developed for irritant contact dermatitis [122].

### 5.14. Sacran

#### 5.14.1. Natural Source, Physicochemical Features, and Bioactive Properties

*Aphanothece sacrum* is a cyanobacterium from which sacran (Figure 1) is extracted [33,82]. This polysaccharide has a high molecular weight (2.35 × 10^7^ g/mol) and presents many carboxylic and sulfuric acid groups. Its sugars are fructose, rhamnose, xylose, arabinose, mannose, glucose, galactose, galactosamine, glucuronic, and galacturonic acids. It affords significant water retention in cells [123].

Sacran has been reported to prevent bacterial and viral invasion, as well as to have the potential to prevent lipids’ absorption and improve intestinal microbiota [124].

#### 5.14.2. Drug Delivery Systems and Pharmacological Activity

This sulfated polysaccharide has been used as basic material in hydrogel films for skin application given its safety and moisturizing effect, with its use being documented in AD patients. This results from its ability to suppress inflammatory cytokines and reduce chemokine mRNA levels. It is indeed a novel biomaterial useful in improving the skin barrier in AD. It has also been reported that it was able to block IL-5, IFN-γ, and TNF-α in an AD mouse model prepared by DNFB stimulation [125]. Ren et al. [123] observed that sacran relieved the symptoms of AD-induced mice, specifically AD score, ear thickness, and IgE release. They also concluded that it inhibits the activation of Th2 cells [123].

Based on this, Wathoni and co-workers developed sacran hydrogel films and studied, among other properties, their skin hydration efficacy [33]. These physically crosslinked sacran hydrogels were prepared by a solvent-casting method and characterized by several techniques. In vivo studies for hydration and for wound healing were carried out using hairless mice and by performing a biopsy punch in the dorsal site of HR-1 mice, respectively. These properties, together with results from the in vivo assays in hairless mice with significant increase in moisture content, show that sacran hydrogels have potential properties as basic biomaterial in AD given their moisturizing and anti-inflammatory effect [33].

### 5.15. Silibinin

#### 5.15.1. Natural Source, Physicochemical Features, and Bioactive Properties

*Silybum marianum* (L.) Gaertn. (Asteraceae family), commonly known as milk thistle or wild artichoke, is the main botanical source of silymarin. Interestingly, silymarin is not an isolated compound, instead is a complex of other important compounds, such as silychristin, silydianin, isosilybin, and silibinin (Figure 1), the latter also known as silybin. The last and most important, silibinin (C_25_H_22_O_10_), is a flavonolignan-type compound formed when the flavononol taxifolin conjugates with coniferyl alcohol, a structural building block mostly found within lignin scaffolds. The structure of silibinin bears several hydroxyl groups, that give the molecule a high antioxidant capacity, as well as the capacity to chelate metals, plus the chromone fragment that enables silibinin to easily react with bases. The main potential of these flavonoid-like compounds is the hepatoprotective effects, which are directly correlated to their high antioxidant activity and membrane stabilizer capacity that avoids lipid peroxidation phenomena [126,127].

#### 5.15.2. Drug Delivery Systems and Pharmacological Activity

The rising interest in the incorporation of silibinin into topical nanaocarriers owes to its recognized antioxidant activity, but more importantly to the anti-inflammatory activity on the skin, arising from the capacity of this flavonoid to suppress NF-ĸB activation as well other proinflammatory genes [128]. Nevertheless, it is worth mentioning that an SLN-enriched gel [129], as well as hydrogels containing pomegranate oil-based NCs loaded with silibinin [128], have been designed as innovative drug delivery systems for irritant contact dermatitis.

A gellan gum/pullulan bilayer film containing silibinin-loaded nanocapsules (NCs) was developed by Gehrcke et al. (2022) [29]. NCs were produced by the method of interfacial deposition of preformed polymer. These silibinin-loaded NCs (content around 98.9%) presented a diameter of 115 ± 3 nm, the PDI was under 0.2, and the ZP was nearly −10 mV. For instance, bilayer films were prepared by the method of two-step solvent casting, using gellan gum as the first polymeric layer, followed by pullulan as the second layer, thus forming a homogeneous bilayer film. According to the results of this study, silibinin was slowly released from the nano-based film and presented high affinity for the cutaneous tissue, thus remaining retained there. In vivo assays were conducted by testing the nano-based formulation on a DNCB-treated mouse model of AD. In comparison either to the silibinin solution alone, the vehicle film itself, or even the ordinary hydrocortisone treatment, the formulation was shown to positively influence the inflammatory and oxidative responses, which was seen in a reduction of the scratching behavior and ear edema. Notwithstanding, the gellan gum/pullulan bilayer film itself, without the silibinin-loaded NCs, also showed some ameliorative effects alone on the DNCB-treated mouse model. Altogether, these data highlight the combination of films and silibinin-loaded NCs as a strategy in AD management, by their anti-inflammatory and antioxidant effects, while encompassing skin hydration and protective properties, in just one formulation [29]. In fact, NCs are vesicular systems consisting of a polymeric membrane surrounding an oily core. Interestingly, as vegetable oils can either function as part of NCs’ scaffolds or as an active ingredient, they have been investigated in this context. NCs’ structural organization favors the encapsulation of lipophilic substances, thus increasing their solubility and therapeutic efficacy, while enhancing drug stability and controlled drug release and decreasing toxicity of active ingredients [18,29,59].

On the other hand, the incorporation of silymarin into a pluronic–lecithin organogel was attempted, which was then tested in patients with AD symptoms [130]. These drug delivery systems are deserving of attention in terms of topical drug administration, as they have a biphasic composition, thus enhancing solubility of poorly solubilizing molecules such as silymarin, plus these formulations also facilitate the penetration of hydrophilic compounds. The authors attempted to produce several formulations with different ratios of pluronic and lecithin, finding optimal concentrations of 20 and 3% for each constituent and thus achieving an optimal silymarin permeation in the ex vivo assays. Furthermore, the designed delivery system improved inflammation symptoms in patients, such as redness and swelling [130].

### 5.16. Triptolide

#### 5.16.1. Natural Source, Physicochemical Features, and Bioactive Properties

*Tripterygium wilfordii* Hook.f., known as thunder god vine or *Lei Gong Teng*, belongs to the Celastraceae botanical family, and it is the natural source from which triptolide was first isolated. Triptolide (C_20_H_24_O_6_) (Figure 1) is an abietane-type diterpene, made up of three epoxy groups and an α, β-unsaturated five-membered lactone. This diterpenoid has been mainly investigated for its antileukemic, anti-inflammatory, immunosuppressive, and anticancer activities. However, the obtention of triptolide directly from medicinal plants is unfeasible given the low concentrations in which is found. However, efforts have been made to develop a lab-scale and industrialized method to synthesize this compound [131].

#### 5.16.2. Drug Delivery Systems and Pharmacological Activity

The anti-inflammatory and immunosuppressive activities of triptolide have been extensively described by several authors, however, not those directly related to skin disorders such as AD. The main findings suggest that such activities are fundamentally related to its suppressive action upon the NF-κB signaling pathway and the IL-17 and IL-6 signals, as well as inhibition of the STAT3-activated signaling pathway. Other evidence shows that triptolide also inhibits the expression of proinflammatory factors [131].

To overcome some challenges related to percutaneous drug delivery, lipidic nanosystems have been proposed as feasible and effective alternatives. In this context, a study on the inclusion of triptolide in NEs was carried out [30]. These nanosystems were prepared by the high-energy emulsification method and demonstrated to provide the best topical drug release and maintenance of concentration. Furthermore, triptolide-loaded NEs improved epidermal lipidic components and keratin characteristics at the epidermal *stratum corneum* layer, refining not only skin hydration but also allowing a better drug permeation. Focusing on the triptolide-containing gels tested in vivo using male SD rats and male ICR mice, in moderate to high dosages, they led to an amelioration of AD-like inflammation and of the erythematous edema of mouse ears. Meanwhile, at the molecular level, these NE-based gels were able to reduce the expression of IFN-γ and IL-4. Once the triptolide-loaded NE gels achieved the best results, they were characterized and were found to be sphere-shaped with a two-layer structure, besides presenting a narrow size distribution of 62.1 ± 9.9 nm and a PDI score of 0.19 ± 0.023 [30].

## 6. Extracts, Oils, and Plant Mixtures Included in Nanotechnology-Based Formulations for the Treatment of AD

### 6.1. Centella asiatica (L.) Urban Extract

#### 6.1.1. General Considerations

*C. asiatica* is particularly rich in triterpenes, namely asiaticoside and madecassoside, and their aglycones, asiatic acid and madecassic acid, respectively. This plant is also characterized by sesquiterpene-rich essential oils, plus other non-volatile compounds such as catechins and the flavonoids kaempferol and quercetin, that are present in plant-derived extracts. However, triterpenes are by far the most important compounds in this recognized medicinal plant. In fact, such compounds have been shown, in several in vitro and in vivo approaches, to act on dermatological diseases such as acne, burns, atopic dermatitis, and wounds via NF-κB, MAPK, and STAT signaling pathways, among others. It is also worth mentioning that *C. asiatica* has been scientifically proved to positively influence different nervous and cognitive functions, namely in Alzheimer’s and Parkinson’s diseases [132].

#### 6.1.2. Drug Delivery Systems and Pharmacological Activity

The establishment of hydrogen bond interactions between phyto-derived molecules and phospholipids forms lipidic-based vesicles called phytosomes. The existence of a double layer phospholipid membrane enables this type of drug delivery system to interact with both polar and non-polar compounds. These characteristics have led their exploration for the delivery of natural compounds with cosmeceutical purposes and for the management of several skin ailments [133], including AD [17].

The antioxidant and anti-inflammatory properties of currently marketed *C. asiatica* phytosomes, either containing extracts or isolated bioactive compounds, are well recognized, such as for wound healing or other skin ailments. However, the effects on AD were only explored by Ho et al. [134]. Therefore, an in vivo phthalic anhydride-induced AD mouse model and in vitro RAW 264.7 murine macrophages were used to study the anti-AD potential of commercially available *C. asiatica* phytosomes. Regarding the in vivo assays, after AD-like lesions were inflicted, 20 µL/cm^2^ of 0.2% and 0.4% of the obtained phytosomes was topically applied on the dorsal skin and ears, for a period of four weeks, three times each. According to the histological analysis, the phytosome decreased hyperkeratosis, proliferation of mast cells, and infiltration of inflammatory cells. Moreover, this formulation not only reduced the expression of NO, iNOS, COX-2, TNF-α, IL-1β, and IgE in vivo but it also reduced the expression of NO, iNOS, and COX-2 in in vitro LPS-stimulated RAW 264.7 macrophage cells. The authors also found that LPS-induced DNA binding activities of NF-κB were affected by phytosome application, thus suggesting a link with the discontinuation of IκBα degradation and consequent decrease in the translocation of p65 and p50 into the nucleus. Overall, the fact that this *C. asiatica* phytosome shows a mechanism of action involving the inhibition of the NF-κB signaling pathway means it has potential in the management of AD [134].

### 6.2. Moutan Cortex and PentaHerbs

#### 6.2.1. General Considerations

Moutan cortex is a traditional Chinese medicine comprising the root bark of *Paeonia* × *suffruticosa* Andrews (Paeoniaceae family). A wide range of phytochemicals have been identified, including flavonoids, tannins, triterpenoids, and glycosylated monoterpenes, despite the predominance and importance of phenolic compounds. This water-insoluble drug presents several pharmacological activities, namely anti-inflammatory, antiallergic, and antioxidant effects, which justifies its use in traditional Chinese medical practices for the treatment of AD [135]. Nevertheless, moutan cortex is also present in the PentaHerbs formula, which consists of a mixture of plant-derived drugs including other traditional Chinese medicines, such as the bark of *Phellodendron chinensis* Schneid. (Rutaceae), the flower of *Lonicera japonica* Thunb. (Caprifoliaceae), the aerial parts of *Mentha haplocalyx* Briq. (Lamiaceae), and the rhizome of *Atractylodes lancea* (Thunb.) DC. (Asteraceae), at the ratio of 2:2:2:1:2 [136]. Moreover, according to traditional Chinese medical practices, this herbal mixture also has antiallergic, anti-inflammatory, antipruritic, and sedative properties, therefore it is extensively used for the treatment of allergic diseases including AD, asthma, and allergic rhinitis. Similarly to moutan cortex, the anti-inflammatory and antiallergic potential of PentaHerbs are suggested to be comparable to corticosteroids’ effects, but without adverse reactions for patients with AD [38].

#### 6.2.2. Drug Delivery Systems and Pharmacological Activity

The effect of PentaHerbs on the release of inflammatory factors from RMPC cells and cytokine production arising from the HMC-1 cell line was investigated to understand how it positively affects AD symptoms [136]. The investigators studied the whole mixture and the effect of different components, finding that moutan cortex and *herba menthae* significantly reduced histamine release and prostaglandin D2 synthesis in the RPMC cell line. Interestingly, moutan cortex was the only component affecting the production of cytokines in HMC-1, while the PentaHerbs formula and the remaining four constituents failed to do so. Overall, the PentaHerbs formula can reduce AD-associated inflammation, and its positive effect has been suggested to be improved if the concentration of moutan cortex is increased [136]. In another investigation, using an in vivo oxazolone-induced dermatitis model, a significant reduction (*p* < 0.05) of ear swelling, epidermis thickening, and eosinophil infiltration in epidermis and dermis, as well as the release of serum IL-12, was found when the aqueous-based extract of the drug was given to the animals by oral or topical administration. In this study, gallic acid, chlorogenic acid, and berberine contents were determined, and the effects of individual compounds were shown. Therefore, both gallic acid and chlorogenic acid inhibited the release of proinflammatory cytokine IL-6 and chemokine CCL7 and CXCL8, when eosinophil–dermal fibroblast co-cultures were submitted to IL-31 and IL-33 treatments, respectively. On the other hand, in the eosinophil culture and eosinophil–dermal fibroblast co-culture, the release of IL-6, CXCL8, CCL2, and CCL7 was also significantly (*p* < 0.05) suppressed by berberine [137].

Recently, investigators developed a dual-responsive hydrogel from thermo-responsive polymer PF127 and two chemically synthesized pH-responsive compounds, N,N,N-trimethyl chitosan (TMC) and polyethylene glycosylated hyaluronic acid (PEG-HA). In this hydrogel, gallic acid was loaded as the active molecule, as it is a major compound found in moutan cortex. In this report, the team showed that the dual-responsive hydrogel (PF127/TMC/PEG-HA) evidenced proper release of gallic acid. Moreover, it was shown that the hydrogel formed by PF127 had improved delivery capacity after adding TMC and PEG-HA [38].

Afterwards, the same team of investigators formulated a dual-responsive hydrogel, using PF127 as the thermo-responsive polymer, while the conjugate made of polysaccharide HA and chitosan oligosaccharide lactate (Chito(oligo)) represented the functional core that is responsive to pH. The polysaccharide-based conjugate was synthesized following carbodiimide chemistry techniques, while the NPs of the conjugate (HA-Ala-Chito(oligo)) were produced by the ultra-sonication methodology. Considering this investigation, gallic acid was selected once again to be the main bioactive compound in the PF127/HA-Ala-Chito(oligo) formulation. According to the characterization of the synthesized hydrogel, it was highly porous and presented an optimal dispersion of the micellar structures, after modification with the nanoconjugate. Such modification resulted in an improvement in the gallic acid delivery behavior. Moreover, the formulation also had its rheological properties improved, as well as mechanical stability and pH responsiveness, after the nanoconjugate was included in the system. The evaluation of the cytotoxicity to HaCaT keratinocytes of the PF127-based formulations presented a cell viability higher than 80.0%, considering drug concentrations ranging between 0.0 and 20.0 μg/mL. From this perspective, the authors further suggest that future research would be necessary to find more harmless biomaterials to successfully use moutan cortex in the treatment of AD, through textile-based transdermal therapy [38].

### 6.3. Eupatorium japonicum Thunb. Extract

#### 6.3.1. General Considerations

*E. japonicum* is a plant species that belongs to the botanical family Asteraceae. In several oriental countries such as Vietnam, Japan, Korea, and China, the leaves are used for the treatment of several gastrointestinal ailments, such as nausea, vomiting, indigestion, and diarrhea [138] Besides that, both leaves and stems are known to be used as analgesic, diuretic, antimicrobial, and vermifuge agents. *E. japonicum* produces an essential oil mainly represented by thymol, and its extracts also have pyrrolizidine alkaloids, namely indicine, amabiline, viridiflorine, echinatine, and rinderine, which are known for their hepatotoxicity and anticancer effects [139]. Moreover, the extracts of this plant are also recognized by their anti-inflammatory potential along with cytotoxic effects, which were attributed to the presence of sesquiterpene lactones [138].

#### 6.3.2. Drug Delivery Systems and Pharmacological Activity

An inflammation-induced human keratinocyte model was used to evaluate the efficacy of gold nanoparticles (AuNPs) loaded with *E. japonicum* flavonoids [39]. Metal-based NPs such as silver NPs (AgNPs) and AuNPs usually lead to an improvement in bioactive performance, an effective entrapment of the drug, and increased delivery capacity, plus high targeting of affected sites, while including a reduced systemic permeation [65]. In fact, AuNPs are suitable nanosystems given their skin and follicular drug delivery abilities and diagnostic and therapeutic applications, so some attempts at their use to treat AD have been made [39].

Briefly, AuNPs were obtained by a reaction between distilled water containing HAuCl_4_•3H_2_O and the plant-derived extract. AuNPs’ characterization showed that they were efficiently synthetized without any impurities, showing a crystalline structure, and they presented a particle size ranging between 31.0 and 149.1 nm with predominant circular, spherical, and polygonal morphologies. *E. japonicum* extract presented as major compounds melilotoside, rutin, hyperoside, nictoflorin, cymaroside, and rhamnetin. According to this recent study, the application of these AuNPs was revealed to be less toxic to the HaCaT cell line, in comparison to the *E. japonicum* extract alone. Furthermore, these NPs suppressed the production of inflammatory cytokines and the production of ROS. These effects were found to be linked with the suppression of both MAPK and NF-κB signaling pathways, thus exposing a possible anti-inflammatory mechanism of action for this extract and formulation and its potential in AD management [39].

### 6.4. Houttuynia cordata Thunb. Extract

#### 6.4.1. General Considerations

*H. cordata* is a perennial herb distributed from Nepal to China and Japan, often used as medicinal plant for the treatment of inflammatory diseases such as AD, but also *herpes simplex* and nasal polyps. There are also reports attesting to its aqueous extract’s antioxidant and anticancer activities [34,40]. The aerial parts contain various types of compounds, with twenty of them already isolated: harmala alkaloids, phenolic acids, chlorogenic acid derivatives, phenolic glycosides, phenylpropanoid derivatives, and flavonoids [40].

#### 6.4.2. Drug Delivery Systems and Pharmacological Activity

To afford good skin permeation of *H. cordata* extracts and enhance their anti-AD activity, Kwon and Kim developed cubosomal and liposomal suspensions. They were prepared using a sonication and film hydration method, respectively. The mean diameters were 231.7 and 273.3 nm for cubosomes (CUB) and LIPs, respectively, and the size distribution varied from 73 to 90 nm and 216 to 300 nm for CUB and 100 to 130 nm and 330 to 470 nm for LIPs. These were analyzed using a ZetaPlus analyzer. In vitro skin permeations were investigated in hairless mouse skin. It was observed that both lipid carriers, especially the CUB-based suspension, enhanced skin permeation of the extract with a decrease in IgE production and IL-4 expression and stimulation of IFN-γ expression. Therefore, they concluded that CUB loaded with *H. cordata* extract had an inhibitory effect on the development of AD-like skin lesions and was efficacious for the treatment of AD [34,140]. Given their capacity to avoid enzymatic degradation, CUBs are nanosystems that are usually developed to encapsulate peptide- and protein-derived drugs. They are produced by promoting emulsification of a lipidic fraction with cubic geometry in water containing NPs, in a liquid state, and with crystalline features [70].

### 6.5. Linseed Oil

#### 6.5.1. General Considerations

Among more than two hundred species included in the genus *Linum* L., the plant *Linum usitatissimum* L. is the oldest one. Known as flax or linseed, it has a high nutritional value: omega-3 fatty acid, such as α-linolenic acid and short chain polyunsaturated fatty acids (PUFAs), soluble and insoluble fibers, phytoestrogen-related lignans, proteins, and different antioxidants. It is found in the international food supply as a functional food. From its dried ripe seeds, a very interesting oil (linseed oil, LSO) is extracted, comprising the following fatty acids: stearic, palmitic, linoleic, oleic, and linolenic [36]. Hence, flaxseed has been studied in diet and disease research given the health benefits linked to some of its bioactive compounds: α-linolenic acid (almost 60%) and lignan secoisolariciresinol diglycoside (SDG) [141].

Besides the edible uses of LSO, it also has several beneficial properties such as anti-inflammatory, antioxidant and analgesic, being used for arthritis, cancer, keratoconjunctivitis, and for several skin complaints. In fact, this plant has been used topically to treat skin diseases such as eczema for many years given its “mucilage”, a substance that soothes and softens the skin [36,142].

#### 6.5.2. Drug Delivery Systems and Pharmacological Activity

Linseed oil helps control inflammation via eicosapentaenoic acid (EPA) that results from the conversion of its main omega-3 fatty acid, α-linolenic acid. EPA works as a competitive inhibitor of the conversion of arachidonic acid into prostaglandin E(2) (PGE2) and leukotriene B(4) (LTB4). Its potent capacity to inhibit histamine and bradykinin has also been reported. This makes it a potent anti-inflammatory agent [35,142,143]. EPA has been identified as an important compound in AD. The human metabolism can transform the linolenic acid present in LSO into EPA [144]. So, in order to assess LSO application as an alternative in AD therapy, new and more effective drug delivery systems were developed for LSO, namely emulsions.

Microemulsions have been used as an important technique to increase drug permeation of the skin, lowering skin irritation and with a higher drug-loading volume. The direct topical use of linseed oil is, however, limited by the low permeation into the stratum corneum. To overcome this, Baboota and co-workers designed a topical submicron microemulsion of linseed. Carbopol 971 was used to improve the microemulsion’s viscosity since this is an oil/water type of emulsion. The particle size and zeta potential revealed an average size of 186 nm with a good size distribution. In vitro skin permeation studies revealed that this microemulsion afforded an enhancement of linseed permeation, hence creating a therapeutic approach for inflammation-based skin diseases [35].

Kildaci and colleagues [36] developed new NE formulations containing LSO and investigated their potential in vitro. NEs, which are delivery systems often used in dermatology given their capacity to improve drug release and skin penetration [145], included LSO using the ultrasonic emulsification method. The LSO-NEs prepared were then analyzed, including the average droplet size, polydispersity index, and zeta potential, among others. In vitro release assays were also carried out. Molecular docking analysis was carried out to determine the binding connections that were most likely to be established between the bioactive compounds of LSO (α-linolenic acid, oleic acid, and linoleic acid) and human leukocyte antigens (HLAs), important players in the immune system activation in AD. The NEs developed had an acceptable droplet size (99.0 nm), PDI (0.14), and ZP (−8.79 mV). Molecular docking analysis showed that α-linolenic acid is the best docked ligand. An appropriate skin permeation was also observed, with 78.4 to 100% of LSO being released at the end of 24 and 48 h, respectively. Kildaci et al.’s results showed that the new LSO-NEs afford a topical skin route for effective AD treatment [36].

### 6.6. Pomegranate Seed Oil

#### 6.6.1. General Considerations

Pomegranate seed oil (PSO) is a vegetable oil obtained from the seeds of *Punica granatum* L. (Lythraceae family) [18]. As it is a vegetable oil, PSO comprises a mixture of other individual molecules with recognized value, therefore it has been studied for their beneficial health effects, namely on chronic diseases, such as cancer, osteoporosis, fatty liver disease, and diabetes, plus the antimicrobial, anti-inflammatory, and immunomodulatory properties [146]. Pomegranate seeds correspond to nearly 10% of the total fruit weight and they have plenty of carbohydrates such as pectin and fibers, besides the presence of vitamins E, C, and K, minerals, as well as phenolic and flavonoid compounds. In addition, pomegranate seeds also contain triterpenoids and phytosterols such as 17-α-estradiol and estriol. Among the fatty acid fraction, there are saturated (ranging from 30 to 35%), monounsaturated (varying between 35 and 37%), diunsaturated (amounts ranging from 25 to 39%) and polyunsaturated (1 to 10%) fatty acids, the latter mainly represented by punicic acid, the major compound found in PSO [147].

#### 6.6.2. Drug Delivery Systems and Pharmacological Activity

The authors of the following study focused their interest on PSO, because it has previously shown interesting pharmacological evidence as an anti-inflammatory and antioxidant vegetable oil, which are key properties for AD management, and because it may function as a main contributor to the construction of the NC scaffold. Therefore, Cervi et al. [18] designed pullulan films loaded with PSO NCs, using the solvent-casting method to prepare the pullulan films and the interfacial precipitation of preformed polymer methodology to produce the NCs. Meanwhile, to have a comparable nanosystem, NEs of PSO were also prepared by the spontaneous emulsification method. In the in vivo assays using DNCB-treated mice, both free PSO and pullulan films containing PSO NCs alleviated AD-like lesions. However, the biochemical analyses suggest that pullulan films loaded with PSO-loaded NCs were the only formulation able to promote alleviation of inflammatory and redox status parameters in the AD-like lesions of the DNCB-treated mouse model. An in vitro safety test revealed that these formulations are safe once they do not provoke skin irritation. Focusing on the characterization of these PSO-containing NCs, these formulations demonstrated an adequate size, with a mean diameter of 181 ± 6 nm, and a PDI under 0.2, while the obtained ZP was around 43.13 ± 0.7 mV. The fabricated pullulan films were characterized as hydrophilic and flexible. In this investigation, NCs were incorporated in polymeric films, because such nanosystems require an embedding matrix that enhances their consistency and dosage form to be topically applied. Among the advantages of such stabilizer films, they provoke few skin irritation side effects and reduce the sticky sensation during topical application and remain for longer periods of time at the affected skin sites and are suitable for the inclusion of hydrophilic solutions. Interestingly, pullulan is also a natural polymer, belonging to the carbohydrate group, and it is obtained from the fermentation of a fungus [18]. It is worth mentioning that PSO has also been used to produce a hydrogel loaded with silibinin, but targeting irritant contact dermatitis instead [128].

### 6.7. Rhus verniciflua Stokes Extract

#### 6.7.1. General Considerations

*Rhus verniciflua* Stokes is an Asian tree, native to China and the Indian subcontinent. There are reports attesting to its benefit to health by improving circulatory problems and blood homeostasis and use as a cathartic, diaphoretic, and antirheumatic [148]. Its extract has revealed important bioactivities such as antibacterial, anti-inflammatory, antiallergic, neuroprotection, and antiosteoporotic. Additionally, its oral intake has proved to be protective against AD. The aqueous extract of *R. verniciflua*’s timber is composed essentially of fustin, gallic acid, fisetin, resorcinol, garbanzol, butein, and sulfuretin [37].

#### 6.7.2. Drug Delivery Systems and Pharmacological Activity

Jiang and Sun conducted an in vivo evaluation using the DNCB-induced AD-like symptom model to study sulfuretin. They concluded that it suppressed the immune response in Th2 cells, ameliorating AD symptoms by targeting the GATA3 pathway in those cells [149]. Jeong and coworkers [37] prepared a topical film of pullulan hydrogel matrix loaded with *R. verniciflua* extract (RVE) and tested its efficacy in vivo, in AD-like models. The AD model adopted was based on the subcutaneous injection of capsaicin in neonates rat pups. Films were prepared by a mixture of pullulan and RVE with an average amount of 0.95% (0.26 mg/film) and resulted in a decrease in mast cells lesions which suggests efficacy against AD.

### 6.8. Tea Tree Oil

#### 6.8.1. General Considerations

Tea tree oil is an essential oil obtained by distillation of the leaves of *Melaleuca alternifolia* (Maiden & Betche) Cheel, which is part of the Myrtaceae family, the same as that of eucalyptus. It is mainly characterized by the presence of oxygenated monoterpene hydrocarbons, as well as monocyclic and bicyclic monoterpenes, of which terpinen-4-ol is the dominant one. Besides that, several other terpenes are found, such *as* γ-terpinene, α-terpinene, 1,8-cineole, p-cymene, terpinolene, α-terpineol, α-pinene, sabinene, aromadendrene, ledene, δ-cadinene, limonene, globulol, and viridiflorol [150].

#### 6.8.2. Drug Delivery Systems and Pharmacological Activity

Terpenoids such as terpinen-4-ol, 𝛼-terpineol, and 1,8-cineole have been suggested to significantly decrease the level of proinflammatory factors, such as TNF-𝛼, IL-1𝛽, IL-8, and IL-10. From this perspective, the potential of this essential oil in the treatment of AD has been explored, by loading it into ETOs [32]. Phosphatidylcholine at 2% and 3% (*w*/*v*) and ethanol at 20%, 30%, and 40% (*w*/*v*) were used to formulate ETOs containing tea tree oil. Optimized ETOs were characterized has having an EE of 76.19 ± 3.26%, a vesicle size of 333.6 nm, and a ZP of −35.3 mV. Afterwards, optimized ETOs were included in a base cream formulated by the phase inversion method. In comparison to the conventional cream, the ETO-based formulation presented a better ex vivo permeation and subsequent deposition at the epidermal and dermal layers, besides not being toxic to keratinocytes in vitro (HaCaT cell line). In addition, according to in vivo assays, inflammatory parameters showed a reduction regarding the severity of clinical score in a BALB/c mice model, as well as a decrease in the infiltration of white blood cells, eosinophils, and IgE antibodies. Besides that, this ETO-based cream may avoid oxidative degradation and improve drug stability and permeation across skin layers. The authors further argued that the easy applicability of the method used to produce this ETO-based formulation may allow it to be scaled up [32].

## 7. Conclusions

Natural products have proved their beneficial effects and advantages in the treatment of several skin diseases, especially when they are used in nanotechnology-based formulations. Therefore, in this paper, studies on the development novel nano-based systems regarding the delivery of natural products, including sixteen isolated compounds, four plant extracts, one plant mixture, and three plant oils, were reviewed, regarding the treatment of AD. However, available data on clinical effectiveness of such nanosystems loaded with natural ingredients are sparse or even non-existent in most cases, as most studies remain at the preclinical research stage and are fundamentally based on single-animal models. Therefore, with this review we expect to prompt new investigations into natural products and drug delivery development, but also to inspire clinicians to evolve additional and robust clinical trials that may attest to the reliability of these approaches, prompting future application in clinical practice. In this sense, we believe this review constitutes a paramount scientific basis to pave new avenues into the management of AD through natural-based healthcare solutions.

## Figures and Tables

**Table 1:** Physicochemical Properties of key nanoformulation-based natural isolated compounds for the treatment of AD.

Natural Isolated Compound	Nanotechnology-Based Formulation	Preparation Approach	EE (%)	PS (nm)	ZP (mV)	PDI	References
Astaxanthin	LIPs	Mixing with high-pressure homogenizer	NA	64.5 ± 2.8	NA	NA	[20]
β-carotene	NFs	Electrospinning	NA	400–800	NA	NA	[21]
Capsaicin	SLNs	Microelmusion method	99	277.4 ± 12.0	NA	0.192 ± 0.095	[13]
Curcumin	SLNs	Microelmusion method	62	493.6 ± 183.90	NA	263 ± 0.043	[13]
	SLN-based gel	Microelmusification with high-speed homogenization method	83.10 ± 2.29	109.2	NA	NA	[22]
	Zein–silk sericin NPs	Antisolvent method	NA	330 to 400	−22 to −25	0.29 to 0.49	[23]
Cynaroside	Hydrogels	Mixing	NA	22,000–26,000	NA	NA	[24]
Dictamnine	Nanocarrier-encapsulated	Using U-SiM bioreactor (ultrasound composite stream-impinging mixer)	93.70	186 ± 30	NA	0.146 ± 0.072	[25]
Epigallocatechin-3-gallate	Gelatin NPs	Self-assembly method	NA	112.5 ± 19.09	23.2 ± 0.5	0.3 ± 0.05	[14]
	Polyethylene glycol-poly lactic-co-glycolic acid -epigallocatechin-3-gallate nanoparticles (PEG-PLGA-EGCG-NPs)	Double emulsion method	86	176.2	−33.3	0.044	[15]
Glycyrrhizic acid	TRAs	Thin film hydration method	66.23 ± 0.61 to 93.10 ± 0.3	56.94 to 270.40	−4.76	0.13 to 1.00	[16]
*Guar gum*	NPs	Acid hydrolysis from guar gum dispersed in water	NA	30–80	−30 ± 5	0.259	[26,27]
Hederagenin	NPs	Emulsion method	NA	10.9	NA	NA	[28]
Piperine	ETO-based cream	Cold method	74.30 ± 3.88	318.1	−32.6	NA	[19]
Quercetin	NLCs	Emulsion evaporation–solidification method	89.95 ± 0.16	215.2	−20.10 ± 1.22	NA	[12]
Resveratrol	SLNs	Microelmusion method	85	271.8 ± 4.0	NA	0.005	[13]
Silibinin	NC-based bilayer film	NCs were prepared by interfacial deposition of preformed polymer method. Films were prepared by two-step solvent-casting method	99	115 ± 3	−10	<0.2	[29]
Triptolide	NE-based gel	High-energy emulsification method	85	62.1 ± 9.9	NA	0.19 ± 0.023	[30]

ETO: Ethosome; LIPs: Liposomes; NA: Not applied; NC: Nanocapsule; NE: Nanoemulsion; NFs: Nanofibers; NLCs: Nanostructured lipid carriers; NPs: Nanoparticles; PDI: Polydispersity index; PS: Particle size; SLNs: Solid lipid nanoparticles; TRAs: Transfersomes; ZP: Zeta potential.

**Table 2:** Key nanoformulation-based natural isolated compounds and their main pharmacological effects.

Natural Isolated Compound	Major Natural Source	Nanotechnology-Based Formulation	Pharmacological Effects	Reference
Astaxanthin	Microalgae, crustaceans, seafood, yeast, fungi, complex plants, birds’ feathers	LIPs	In vivo: STAT3 and NF-κB inhibition	[20]
β-carotene	Plants, marine algae, fungi, and bacteria	NFs	In vitro: very slow degradability rate and gradual release of beta-carotene	[21]
Curcumin	*Curcuma longa* L.	SLNs	In vitro: ↓ IL-6. No cytotoxic effects for NCTC 2544 and THP-1 monocytes differentiated into M2 macrophages	[13]
		SLN-based gel	In vivo: ↓ TNF-α and IL-6. ↑ healing lesions and skin hydration. Improved redox status (↑ GSH and catalase; MDA ↓). Ex vivo: ↑ deep penetration into the dermis.	[22]
		Zein–silk sericin NPs	Ex vivo: ↑ deep penetration and skin permeability. In vitro: ↓ NF-κBp65, inflammatory cytokines, and chemokines in HaCaT keratinocytes	[23]
Cynaroside	*Bidens tripartita* L., *Verbascum lychnitis* L., *Elsholtiza bodinieri* Vaniot	Hydrogels	In vivo: ↓ skin and tissue inflammation and inflammatory infiltrates; ↓ number of T and mast cells and histiocytes; hindered the overexpression of cytokines and IgE levels	[24]
Dictamnine	*Dictamnus dasycarpus* Turcz.	Nanocarrier-encapsulated	In vivo: ↓ thymic stromal lymphopoietin (TSLP), IL-1β and TNF-α expression; improvement of skin inflammation	[25]
Epigallocatechin-3-gallate	*Vitis vinifera* L.	Gelatin NPs	In vivo: ↑ skin absorbance and no side effects. In vitro: ↓ IL-6 and IL-8 in LPS-inflamed WS1 dermal fibroblasts	[14]
		Polyethylene glycol-poly lactic-co-glycolic acid -epigallocatechin-3-gallate nanoparticles (PEG-PLGA-EGCG-NPs)	In vivo: ↓ ear and skin thickness, dermatitis score, and scratching behavior. Restoration of redox status (↑ SOD, GSH, and T-AOC). ↓ Th1 (IFN-g and TNF-α), Th2 (IL-4 and IL-6), and Th17 (IL-17A) cytokines. Supression of RIP1, RIP3, MLKL, p-p38, ERK1, and ERK2	[15]
		Epigallocatechin gallate/L-ascorbic acid-loaded poly-γ-glutamate microneedles (EGCG/AA-loaded-γ-PGA MNs)	In vivo: ↓ dermatitis score, mast cell infiltration, IFN-γ expression, Th2 cytokine secretion, IgE, and histamine	[31]
Glycyrrhizic acid	*Glycyrrhiza glabra* L.	TRAs	In vivo: ↓ erythema signs and scratching behavior. Ex vivo: ↓ permeation and ↑ skin deposition	[16]
Guar gum	*Cyamopsis tetragonoloba* (L.) Taub.	NPs	In vitro: successful wound-healing effect; In vivo: ↓ AD symptoms, such as redness and epidermal thickness; ↓ serum IgE levels, total counts for blood cells, skin cells, eosinophils, macrophages, and neutrophils	[26]
Hederagenin	*Sapindus saponaria* L., *Lonicera japonica* Thunb.	NPs	In vivo: Dose-dependent inhibition of IFN-γ, TNF-α, IL-4, IL-6, IL-17, and thymic stromal lymphopoietin (TSLP); ↓ mast cell infiltration, epidermal and dermal thickness of mouse skin; relieved lumping lymph nodes	[28]
Piperine	*Piper nigrum* L.	ETO-based cream	In vivo: ↓ ear and skin thickness, skin severity, white blood cells, granulocytes, and IgE. Ex vivo: penetration and deposition. In vitro: no cytotoxic effects in HaCaT keratinocytes	[32]
Quercetin	Present in several food products such as fruits and vegetables	NLCs	In vivo: ↓ inflammation symptoms. In vitro: ↑ percutaneous permeabilization and retention at the dermis and epidermis	[12]
Resveratrol	*Vitis vinifera* L.	SLNs	In vitro: ↓ IL-6 and MCP-1. No cytotoxic effects for NCTC 2544 and THP-1 monocytes differentiated into M2 macrophages	[13]
Sacran	*Aphanothece sacrum* (Sur.) Okada	Hydrogel films	In vivo: ↑ moisture content	[33]
Silibinin	*Silybum marianum* L.	NC-based bilayer film	In vivo: ↓ oxidative and inflammatory markers, ↓ scratching behavior and ear edema, ↑ skin hydration. Ex vivo: controlled dug release, ↑ drug retention. In vitro: ↑ antioxidant potential	[29]
Triptolide	*Tripterygium wilfordii* Hook. F.	NE-based gel	In vivo: ↓ ear edema. ↓ IFN-γ and IL-4. Ex vivo: ↑ deep penetration and percutaneous delivery	[30]

ETOs: Ethosome; LIPs: Liposomes; NC: Nanocapsule; NE: Nanoemulsion; NFs: Nanofibers; NLCs: Nanostructured lipid carriers; NPs: Nanoparticles; SLNs: Solid lipid nanoparticles; TRAs: Transfersomes.

**Table 3:** Physicochemical properties of key nanoformulation-based extracts and oils for the treatment of AD.

Extract/Oil/Mixture	Nanotechnology-Based Formulation	Preparation Approach	EE (%)	PS (nm)	ZP (mV)	PDI	Reference
*Houttuynia cordata* Thunb.	CUBs	Sonication	NA	231.7	NA	NA	[34]
	LIP suspensions	Film hydration method	NA	273.3	NA	NA	[34]
Linseed oil	Microemulsion	NA	NA	186	NA	NA	[35]
	NEs	Ultrasonication method	NA	99.02 ± 1.06	−8.79 ± 0.034	0.14 ± 0.020	[36]
Pomegranate seed oil	NC-based film	Solvent-casting method to prepare the pullulan films and the interfacial precipitation of preformed polymer methodology to produce NCs	NA	181 ± 6	43.13 ± 0.7	<0.2	[18]
*Rhus verniciflua* Stokes	Hydrogel	Mixture stirred for complete solubilization at RT and then cast onto glass plates of 4 mm thickness	0.95	NA	NA	NA	[37]
Tea tree oil	ETO-based cream	ETOs were obtained by mixing of reagents and subsequent sonication. Creams were obtained by phase inversion method	76.19 ± 3.26	333.6	−35.3	NA	[19]

CUBs: Cubosomes; ETO: Ethosome; LIP: Liposome; NA: Not applicable; NC: Nanocapsule; NEs: Nanoemulsions; PDI: Polydispersity index; PS: Particle size; ZP: Zeta potential.

**Table 4:** Key nanoformulation-based plant mixtures, oils, and extracts and their main pharmacological effects.

Extract/Oil/Mixture	Major Compounds	Nanotechnology-Based Formulation	Pharmacological Effects	References
*Centella asiatica* (L.) Urban	Triterpenes, namely asiaticoside and madecassoside, and their aglycones	Phytosome	In vivo: ↓ hyperkeratosis, proliferation of mast cells and infiltration of inflammatory cells. ↓ expression of iNOS, COX-2, NF-κB, TNF-α, IL-1β, and IgE. In vitro: ↓ NO, iNOS, and COX-2 in LPS-stimulated RAW 264.7 macrophages. ↓ LPS-induced DNA-binding activities of NF-κB	[17]
Cortex Moutan	Plant mixture. Gallic acid	PF127/HA-Ala-Chito(oligo)-based hydrogel	In vitro: cell viability of >80.0% in HaCaT keratinocytes, considering concentrations ranging between 0.0 and 20.0 μg/mL	[38]
*Eupatorium japonicum* Thunb.	Flavonoids, namely melilotoside, rutin, hyperoside, nictoflorin, cymaroside, and rhamnetin	AuNPs	In vitro: suppression of MAPK and nuclear factor-κB signaling pathways. ↓ RANTES, TARC, CTACK, IL-6, and IL-8. ↓ production of ROS	[39]
*Houttuynia cordata* Thunb.	Harmala alkaloids, phenolic acids, chlorogenic acid derivatives, phenolic glycosides, phenylpropanoid derivatives, and flavonoids	CUB and LIP suspensions	In vivo: ↑ skin permeation of the extract; ↓ IgE production and IL-4 expression; ↑ IFN-γ expression	[34,40]
Linseed oil	Omega-3 fatty acid, such as α-linolenic acid and short chain polyunsaturated fatty acids (PUFAs)	Microemulsion	In vitro: ↑ linseed permeation	[35]
	Omega-3 fatty acid, such as α-linolenic acid and short chain polyunsaturated fatty acids (PUFAs)	NEs	In vitro: adequate linseed permeation	[36]
Pomegranate seed oil	Complex mixture rich in punicic acid	NC-based film	In vivo: ↓ AD-like skin injury, ↑ oxidative defenses, ↓ hypernocipetive behavior. In vitro: absence of irritation	[18]
*Rhus verniciflua* Stokes	Fustin, gallic acid, fisetin, resorcinol, garbanzol, butein, and sulfuretin	Hydrogel	In vivo: ↓ mast cell lesions	[37]
Tea tree oil	Terpinen-4-ol is the major monoterpene in this essential oil	ETO-based cream	In vivo: ↓ severity of clinical score, infiltration of white blood cells, eosinophils, and IgE antibodies. Ex vivo: ↑ Drug permeation and retention. In vitro: absence of cytotoxic effects in HaCaT keratinocytes	[19]

AuNPs: Gold nanoparticles; CUB: Cubosome; ETO: Ethosome; LIP: Liposome; NC: Nanocapsule; NPs: Nanoparticles; NEs: Nanoemulsions.

## References

[1] Marques M.P., Mendonça L., Neves B.G., Varela C., Oliveira P., Cabral C. (2023). Exploring Iberian Peninsula Lamiaceae as Potential Therapeutic Approaches in Wound Healing. Pharmaceuticals.

[2] Zaid N.A.M., Sekar M., Bonam S.R., Gan S.H., Lum P.T., Begum M.Y., Rani N.N.I.M., Vaijanathappa J., Wu Y.S., Subramaniyan V. (2022). Promising Natural Products in New Drug Design, Development, and Therapy for Skin Disorders: An Overview of Scientific Evidence and Understanding Their Mechanism of Action. Drug Des. Dev. Ther..

[3] Archer C.B. (2021). Atopic Dermatitis. Medicine.

[4] Langan S.M., Irvine A.D., Weidinger S. (2020). Atopic Dermatitis. Lancet.

[5] Song A., Lee S.E., Kim J.H. (2022). Immunopathology and Immunotherapy of Inflammatory Skin Diseases. Immune Netw..

[6] Ujiie H., Rosmarin D., Schön M.P., Ständer S., Boch K., Metz M., Maurer M., Thaci D., Schmidt E., Cole C. (2022). Unmet Medical Needs in Chronic, Non-Communicable Inflammatory Skin Diseases. Front. Med..

[7] Paiva-Santos A.C., Gama M., Peixoto D., Sousa-Oliveira I., Ferreira-Faria I., Zeinali M., Abbaspour-Ravasjani S., Mascarenhas-Melo F., Hamishehkar H., Veiga F. (2022). Nanocarrier-Based Dermopharmaceutical Formulations for the Topical Management of Atopic Dermatitis. Int. J. Pharm..

[8] Sharma S., Naura A.S. (2020). Potential of Phytochemicals as Immune-Regulatory Compounds in Atopic Diseases: A Review. Biochem. Pharmacol..

[9] Wu S., Pang Y., He Y., Zhang X., Peng L., Guo J., Zeng J. (2021). A Comprehensive Review of Natural Products against Atopic Dermatitis: Flavonoids, Alkaloids, Terpenes, Glycosides and Other Compounds. Biomed. Pharmacother..

[10] Qadir A., Ullah S.N.M.N., Jahan S., Ali A., Khan N. (2022). Drug Delivery of Natural Products through Nano-carriers for Effective Vitiligo Therapy: A Compendia Review. J. Cosmet. Dermatol..

[11] Xie J., Huang S., Huang H., Deng X., Yue P., Lin J., Yang M., Han L., Zhang D.K. (2021). Advances in the Application of Natural Products and the Novel Drug Delivery Systems for Psoriasis. Front. Pharmacol..

[12] Guo C.Y., Yang C.F., Li Q.L., Tan Q., Xi Y.W., Liu W.N., Zhai G.X. (2012). Development of a Quercetin-Loaded Nanostructured Lipid Carrier Formulation for Topical Delivery. Int. J. Pharm..

[13] Cassano R., Serini S., Curcio F., Trombino S., Calviello G. (2022). Preparation and Study of Solid Lipid Nanoparticles Based on Curcumin, Resveratrol and Capsaicin Containing Linolenic Acid. Pharmaceutics.

[14] Drew V.J., Huang H., Tsai Z.-H., Tsai H., Tseng C.-L. (2017). Preparation of Gelatin/Epigallocatechin Gallate Self-Assembly Nanoparticles for Transdermal Drug Delivery. J. Polym. Res..

[15] Han M., Wang X., Wang J., Lang D., Xia X., Jia Y., Chen Y. (2022). Ameliorative Effects of Epigallocatechin-3-Gallate Nanoparticles on 2,4-Dinitrochlorobenzene Induced Atopic Dermatitis: A Potential Mechanism of Inflammation-Related Necroptosis Mengguo. Front. Nutr..

[16] Chauhan S., Gulati N., Nagaich U. (2018). International Journal of Polymeric Materials and Fabrication and Evaluation of Ultra Deformable Vesicles for Atopic Dermatitis as Topical Delivery. Int. J. Polym. Mater. Polym. Biomater..

[17] Park J.H., Yeo I.J., Han J.H., Suh J.W., Lee H.P., Hong J.T. (2018). Anti-Inflammatory Effect of Astaxanthin in Phthalic Anhydride-Induced Atopic Dermatitis Animal Model. Exp. Dermatol..

[18] Cervi V.F., Saccol C.P., Sari M.H.M., Martins C.C., da Rosa L.S., Ilha B.D., Soares F.Z., Luchese C., Wilhelm E.A., Cruz L. (2021). Pullulan Film Incorporated with Nanocapsules Improves Pomegranate Seed Oil Anti-Inflammatory and Antioxidant Effects in the Treatment of Atopic Dermatitis in Mice. Int. J. Pharm..

[19] Kumar P., Sharma D.K., Ashawat M.S. (2021). Development of Phospholipids Vesicular Nanocarrier for Topical Delivery of Tea Tree Oil in Management of Atopic Dermatitis Using BALB/c Mice Model. Eur. J. Lipid Sci. Technol..

[20] Lee Y.S., Jeon S.H., Ham H.J., Lee H.P., Song M.J., Hong J.T. (2020). Improved Anti-Inflammatory Effects of Liposomal Astaxanthin on a Phthalic Anhydride-Induced Atopic Dermatitis Model. Front. Immunol..

[21] Semnani D., Nasari M., Fakhrali A. (2018). PCL Nanofibers Loaded with Beta-Carotene: A Novel Treatment for Eczema. Polym. Bull..

[22] Saini K., Modgill N., Singh K.K. (2022). Tetrahydrocurcumin Lipid Nanoparticle Based Gel Promotes Penetration into Deeper Skin Layers and Alleviates Atopic Dermatitis in 2,4-Dinitrochlorobenzene (DNCB) Mouse Model. Nanomaterials.

[23] Zhu J.J., Tang C.H., Luo F.C., Yin S.W., Yang X.Q. (2022). Topical Application of Zein-Silk Sericin Nanoparticles Loaded with Curcumin for Improved Therapy of Dermatitis. Mater. Today Chem..

[24] Szekalska M., Sosnowska K., Tomczykowa M., Winnicka K., Kasacka I., Tomczyk M. (2020). In Vivo Anti-Inflammatory and Anti-Allergic Activities of Cynaroside Evaluated by Using Hydrogel Formulations. Biomed. Pharmacother..

[25] Lin C.-Y., Hsieh Y.-T., Chan L.Y., Yang T.-Y., Maeda T., Chang T.-M., Huang H.-C. (2021). Dictamnine Delivered by PLGA Nanocarriers Ameliorated Inflammation in an Oxazolone-Induced Dermatitis Mouse Model. J. Control. Release.

[26] Ghosh N., Mitra S., Banerjee E.R. (2018). Therapeutic Effects of Topically-Administered Guar Gum Nanoparticles in Oxazolone-Induced Atopic Dermatitis in Mice. Biomed. Res. Ther..

[27] Ghosh S.K., Abdullah F., Mukherjee A. (2015). Fabrication and Fluorescent Labeling of Guar Gum Nanoparticles in a Surfactant Free Aqueous Environment. Mater. Sci. Eng. C.

[28] Lee K.-J., Ratih K., Kim G.-J., Lee Y.-R., Shin J.-S., Chung K.-H., Choi E.-J., Kim E.-K., An J.H. (2022). Immunomodulatory and Anti-Inflammatory Efficacy of Hederagenin-Coated Maghemite (γ-Fe_2_O_3_) Nanoparticles in an Atopic Dermatitis Model. Colloids Surf. B Biointerfaces.

[29] Gehrcke M., Martins C.C., de Bastos Brum T., da Rosa L.S., Luchese C., Wilhelm E.A., Soares F.Z.M., Cruz L. (2022). Novel Pullulan/Gellan Gum Bilayer Film as a Vehicle for Silibinin-Loaded Nanocapsules in the Topical Treatment of Atopic Dermatitis. Pharmaceutics.

[30] Yang M., Gu Y., Yang D., Tang X., Liu J. (2017). Development of Triptolide-Nanoemulsion Gels for Percutaneous Administration: Physicochemical, Transport, Pharmacokinetic and Pharmacodynamic Characteristics. J. Nanobiotechnol..

[31] Chiu Y., Wu Y., Hung J., Chen M. (2021). Epigallocatechin Gallate/L-Ascorbic Acid–Loaded Poly-γ-Glutamate Microneedles with Antioxidant, Anti-Inflammatory, and Immunomodulatory Effects for the Treatment of Atopic Dermatitis. Acta Biomater..

[32] Kumar P., Sharma D.K., Ashawat M.S. (2021). Topical Creams of Piperine Loaded Lipid Nanocarriers for Management of Atopic Dermatitis: Development, Characterization, and in Vivo Investigation Using BALB/c Mice Model. J. Liposome Res..

[33] Wathoni N., Motoyama K., Higashi T., Okajima M., Kaneko T., Arima H. (2016). Physically Crosslinked-Sacran Hydrogel Films for Wound Dressing Application. Int. J. Biol. Macromol..

[34] Kwon T.K., Kim J.C. (2014). In Vitro Skin Permeation and Anti-Atopic Efficacy of Lipid Na- Nocarriers Containing Water Soluble Extracts of Houttuynia Cordata. Drug Dev. Ind. Pharm..

[35] Baboota S., Rahman M.U., Kumar A., Sharma S., Sahni J., Ali J. (2012). Submicron Size Formulation of Linseed Oil Containing Omega-3 Fatty Acid for Topical Delivery. J. Dispers. Sci. Technol..

[36] Kildaci I., Budama-Kilinc Y., Kecel-Gunduz S., Altuntas E. (2021). Linseed Oil Nanoemulsions for Treatment of Atopic Dermatitis Disease: Formulation, Characterization, in Vitro and in Silico Evaluations. J. Drug Deliv. Sci. Technol..

[37] Jeong J.H., Back S.K., An J.H., Lee N., Kim D., Na C.S., Jeong Y., Han S.Y. (2019). Topical Film Prepared with Rhus Verniciflua Extract-Loaded Pullulan Hydrogel for Atopic Dermatitis Treatment. J. Biomed. Mater. Res. Part B Appl. Biomater..

[38] Chatterjee S., Hui P.C., Wat E., Kan C., Leung P., Wang W. (2020). Drug Delivery System of Dual-Responsive PF127 Hydrogel with Polysaccharide-Based Nano-Conjugate for Textile-Based Transdermal Therapy. Carbohydr. Polym..

[39] Xu X.Y., Moon S., Kim J., Kim W.J., Kim Y., Kim H. (2022). Structural Properties and Anti-Dermatitis Effects of Fl Avonoids-Loaded Gold Nanoparticles Prepared by Eupatorium Japonicum. Front. Pharmacol..

[40] Ahn J., Kim J. (2016). Chemical Constituents from Houttuynia Cordata. Planta Med..

[41] Wollenberg A., Kinberger M., Arents B., Aszodi N., Avila Valle G., Barbarot S., Bieber T., Brough H.A., Pinton P.C., Christen-Zäch S. (2022). European Guideline (EuroGuiDerm) on Atopic Eczema: Part I—Systemic Therapy. J. Eur. Acad. Dermatol. Venereol..

[42] Wollenberg A., Kinberger M., Arents B., Aszodi N., Valle G.A., Barbarot S., Bieber T., Brough H.A., Pinton P.C., Christen-Zäch S. (2022). European Guideline (EuroGuiDerm) on Atopic Eczema—Part II: Non-Systemic Treatments and Treatment Recommendations for Special AE Patient Populations. J. Eur. Acad. Dermatol. Venereol..

[43] Hamidi M., Azadi A., Rafiei P. (2008). Hydrogel Nanoparticles in Drug Delivery. Adv. Drug Deliv. Rev..

[44] Jeevanandam J., Chan Y.S., Danquah M.K. (2016). Nano-Formulations of Drugs: Recent Developments, Impact and Challenges. Biochimie.

[45] Mazayen Z.M., Ghoneim A.M., Elbatanony R.S., Basalious E.B., Bendas E.R. (2022). Pharmaceutical Nanotechnology: From the Bench to the Market. Futur. J. Pharm. Sci..

[46] Halwani A.A. (2022). Development of Pharmaceutical Nanomedicines: From the Bench to the Market. Pharmaceutics.

[47] Ratemi E., Shaik A.S., Al Faraj A., Halwani R. (2016). Alternative Approaches for the Treatment of Airway Diseases: Focus on Nanoparticle Medicine. Clin. Exp. Allergy.

[48] Scientific Committee on Emerging and Newly Identified Health Risks (SCENIHR) (2006). The Appropriateness of Existing Methodologies to Assess the Potential Risks Associated with Engineered and Adventitious. https://health.ec.europa.eu/scientific-committees/former-scientific-committees/scientific-committee-emerging-and-newly-identified-health-risks-scenihr_en.

[49] Jong W., Borm P. (2008). Drug Delivery and Nanoparticles: Applications and Hazards. Int. J. Nanomed..

[50] Lotfipour F., Shahi S., Farjami A., Salatin S., Mahmoudian M., Dizaj S.M. (2021). Safety and Toxicity Issues of Therapeutically Used Nanoparticles from the Oral Route. Biomed Res. Int..

[51] Doktorovová S., Kovačević A.B., Garcia M.L., Souto E.B. (2016). Preclinical Safety of Solid Lipid Nanoparticles and Nanostructured Lipid Carriers: Current Evidence from In Vitro and In Vivo Evaluation. Eur. J. Pharm. Biopharm..

[52] Wolfram J., Zhu M., Yang Y., Shen J., Gentile E., Paolino D., Fresta M., Nie G., Chen C., Shen H. (2015). Safety of Nanoparticles in Medicine. Curr. Drug Targets.

[53] Fassett R.G., Coombes J.S. (2011). Astaxanthin: A Potential Therapeutic Agent in Cardiovascular Disease. Mar. Drugs.

[54] Fakhri S., Abbaszadeh F., Dargahi L., Jorjani M. (2018). Astaxanthin: A Mechanistic Review on Its Biological Activities and Health Benefits. Pharmacol. Res..

[55] Alugoju P., Swamy V.K.D.K., Anthikapalli N.V.A., Tencomnao T. (2022). Health Benefits of Astaxanthin against Age-Related Diseases of Multiple Organs: A Comprehensive Review. Crit. Rev. Food Sci. Nutr..

[56] Hong L., Zhou C.-L., Chen F.-P., Han D., Wang C.-Y., Li J.-X., Chi Z., Liu C.-G. (2017). Development of a Carboxymethyl Chitosan Functionalized Nanoemulsion Formulation for Increasing Aqueous Solubility, Stability and Skin Permeability of Astaxanthin Using Low-Energy Method. J. Microencapsul..

[57] Eren B., Tanrıverdi S.T., Köse F.A., Özer Ö. (2019). Antioxidant Properties Evaluation of Topical Astaxanthin Formulations as Anti-aging Products. J. Cosmet. Dermatol..

[58] Geng Q., Zhao Y., Wang L., Xu L., Chen X., Han J. (2020). Development and Evaluation of Astaxanthin as Nanostructure Lipid Carriers in Topical Delivery. AAPS PharmSciTech.

[59] Hemrajani C., Negi P., Parashar A., Gupta G., Jha N.K., Singh S.K., Chellappan D.K., Dua K. (2022). Overcoming Drug Delivery Barriers and Challenges in Topical Therapy of Atopic Dermatitis: A Nanotechnological Perspective. Biomed. Pharmacother..

[60] Choo W.-T., Teoh M.-L., Phang S.-M., Convey P., Yap W.-H., Goh B.-H., Beardall J. (2020). Microalgae as Potential Anti-Inflammatory Natural Product against Human Inflammatory Skin Diseases. Front. Pharmacol..

[61] Rühl R., Taner C., Schweigert F.J., Wahn U., Grüber C. (2010). Serum Carotenoids and Atopy among Children of Different Ethnic Origin Living in Germany. Pediatr. Allergy Immunol..

[62] Kake T., Imai M., Takahashi N. (2019). Effects of Β-carotene on Oxazolone-induced Atopic Dermatitis in Hairless Mice. Exp. Dermatol..

[63] Takahashi N., Kake T., Hasegawa S., Imai M. (2019). Effects of Post-Administration of β-Carotene on Diet-Induced Atopic Dermatitis in Hairless Mice. J. Oleo Sci..

[64] Arslan E., Garip I.C., Gulseren G., Tekinay A.B., Guler M.O. (2014). Bioactive Supramolecular Peptide Nanofibers for Regenerative Medicine. Adv. Healthc. Mater..

[65] Paiva-santos A.C., Mascarenhas-melo F., Coimbra S.C., Pawar K.D., Peixoto D., Chá-chá R., Araujo A.R.T.S., Pinto S., Veiga F., Cláudia A. (2021). Expert Opinion on Drug Delivery Nanotechnology-Based Formulations toward the Improved Topical Delivery of Anti-Acne Active Ingredients. Expert Opin. Drug Deliv..

[66] Basith S., Cui M., Hong S., Choi S. (2016). Harnessing the Therapeutic Potential of Capsaicin and Its Analogues in Pain and Other Diseases. Molecules.

[67] Kumar V., Bhatt V., Kumar N. (2018). Amides from Plants: Structures and Biological Importance.

[68] Rios M.Y., Olivo H.F. (2014). Natural and Synthetic Alkamides: Applications in Pain Therapy.

[69] Ghiasi Z., Esmaeli F., Aghajani M., Ghazi-khansari M. (2019). Enhancing Analgesic and Anti-Inflammatory Effects of Capsaicin When Loaded into Olive Oil Nanoemulsion: An In Vivo Study. Int. J. Pharm..

[70] Souto E.B., Dias-ferreira J., Oliveira J., Sanchez-lopez E., Martins-gomes C., Silva M. (2019). Trends in Atopic Dermatitis—From Standard Pharmacotherapy to Novel Drug Delivery Systems. Int. J. Mol. Sci..

[71] Wang X.-R., Gao S.-Q., Niu X.-Q., Li L.-J., Ying X.-Y., Hu Z.-J., Gao J.-Q. (2017). Capsaicin-Loaded Nanolipoidal Carriers for Topical Application: Design, Characterization, and In Vitro/In Vivo Evaluation. Int. J. Nanomed..

[72] Raza K., Shareef M.A., Singal P., Sharma G., Negi P., Prakash O. (2014). Lipid-Based Capsaicin-Loaded Nano-Colloidal Biocompatible Topical Carriers with Enhanced Analgesic Potential and Decreased Dermal Irritation. J. Liposome Res..

[73] Ghosalkar S., Prabha M., Padmini S. (2021). Emerging Topical Drug Delivery Approaches for the Treatment of Atopic Dermatitis. J. Cosmet. Dermatol..

[74] Vollono L., Falconi M., Gaziano R., Iacovelli F., Dika E., Terracciano C., Bianchi L., Campione E. (2019). Potential of Curcumin in Skin Disorders. Nutrients.

[75] Moon P., Jeong H., Kim H. (2013). Down-Regulation of Thymic Stromal Lymphopoietin by Curcumin. Pharmacol. Rep..

[76] Journal A.I., Shrotriya S., Ranpise N., Satpute P., Vidhate B. (2017). Skin Targeting of Curcumin Solid Lipid Nanoparticles-Engrossed Topical Gel for the Treatment of Pigmentation and Irritant Contact Dermatitis. Artif. Cells Nanomed. Biotechnol..

[77] Ternullo S., Gagnat E., Julin K., Johannessen M., Basnet P. (2019). European Journal of Pharmaceutics and Biopharmaceutics Liposomes Augment Biological Bene Fi Ts of Curcumin for Multitargeted Skin Therapy. Eur. J. Pharm. Biopharm..

[78] Zou Y., Zhang M., Zhang T., Wu J., Wang J., Liu K., Zhan N. (2018). Antioxidant and Anti-Inflammatory Activities of Cynaroside from *Elsholtiza bodinieri*. Nat. Prod. Commun..

[79] Baskar A.A., Ignacimuthu S., Michael G.P., Al Numair K. (2010). Cancer Chemopreventive Potential of Luteolin-7-O-Glucoside Isolated from *Ophiorrhiza mungos* Linn. Nutr. Cancer.

[80] Tian Y., Sun L.-M., Liu X.-Q., Li B., Wang Q., Dong J.-X. (2010). Anti-HBV Active Flavone Glucosides from *Euphorbia humifusa* Willd. Fitoterapia.

[81] Palombo R., Savini I., Avigliano L., Madonna S., Cavani A., Albanesi C., Mauriello A., Melino G., Terrinoni A. (2016). Luteolin-7-Glucoside Inhibits IL-22/STAT3 Pathway, Reducing Proliferation, Acanthosis, and Inflammation in Keratinocytes and in Mouse Psoriatic Model. Cell Death Dis..

[82] Barbosa A.I., Torres T., Lima S.A.C., Reis S. (2021). Hydrogels: A Promising Vehicle for the Topical Management of Atopic Dermatitis. Adv. Ther..

[83] Qing W., Wang Y., Li H., Ma F., Zhu J., Liu X. (2017). Preparation and Characterization of Copolymer Micelles for the Solubilization and In Vitro Release of Luteolin and Luteoloside. AAPS PharmSciTech.

[84] Qing W., Wang Y., Wang Y., Zhao D., Liu X., Zhu J. (2016). The Modified Nanocrystalline Cellulose for Hydrophobic Drug Delivery. Appl. Surf. Sci..

[85] Gao X., Zhao P.-H., Hu J.-F. (2011). Chemical Constituents of Plants from the Genus Dictamnus. Chem. Biodivers..

[86] Chang T.-M., Yang T.-Y., Niu Y.-L., Huang H.-C. (2018). The Extract of D. Dasycarpus Ameliorates Oxazolone-Induced Skin Damage in Mice by Anti-Inflammatory and Antioxidant Mechanisms. Antioxidants.

[87] Yang B., Lee H.-B., Kim S., Park Y.C., Kim K., Kim H. (2017). Decoction of *Dictamnus Dasycarpus* Turcz. Root Bark Ameliorates Skin Lesions and Inhibits Inflammatory Reactions in Mice with Contact Dermatitis. Pharmacogn. Mag..

[88] Yang N., Shao H., Deng J., Yang Y., Tang Z., Wu G., Liu Y. (2023). Dictamnine Ameliorates Chronic Itch in DNFB-Induced Atopic Dermatitis Mice via Inhibiting MrgprA3. Biochem. Pharmacol..

[89] Mokra D., Joskova M., Mokry J. (2023). Therapeutic Effects of Green Tea Polyphenol (-)-Epigallocatechin-3-Gallate (EGCG) in Relation to Molecular Pathways Controlling Inflammation, Oxidative Stress, and Apoptosis. Int. J. Mol. Sci..

[90] Noh S.U., Cho E.A., Kim H.O., Park Y.M. (2008). Epigallocatechin-3-Gallate Improves *Dermatophagoides pteronissinus* Extract-Induced Atopic Dermatitis-like Skin Lesions in NC/Nga Mice by Suppressing Macrophage Migration Inhibitory Factor. Int. Immunopharmacol..

[91] Aljuffali I.A., Hung C., Shih L., Yang C., Alalaiwe A., You J. (2022). Nanoencapsulation of Tea Catechins for Enhancing Skin Absorption and Therapeutic Efficacy. AAPS PharmSciTech.

[92] Nascimento M.H.M.D., de Araújo D.R. (2022). Exploring the Pharmacological Potential of Glycyrrhizic Acid: From Therapeutic Applications to Trends in Nanomedicine. Futur. Pharmacol..

[93] Kowalska A. (2019). 18 b-Glycyrrhetinic Acid: Its Core Biological Properties and Dermatological Applications. Int. J. Cosmet. Sci..

[94] Wang Y., Zhang Y., Peng G., Han X. (2018). International Immunopharmacology Glycyrrhizin Ameliorates Atopic Dermatitis-like Symptoms through Inhibition of HMGB1. Int. Immunopharmacol..

[95] Barone A., Chiara M., Cilurzo F., Locatelli M., Iannotta D., Di L., Celia C., Paolino D. (2020). Ammonium Glycyrrhizate Skin Delivery from Ultradeformable Liposomes: A Novel Use as an Anti-inflammatory Agent in Topical Drug Delivery. Colloids Surf. B Biointerfaces.

[96] Quan W., Kong S., Ouyang Q., Tao J., Lu S., Huang Y., Li S., Luo H. (2021). Use of 18β-Glycyrrhetinic Acid Nanocrystals to Enhance Anti-Inflammatory Activity by Improving Topical Delivery. Colloids Surf. B Biointerfaces.

[97] Zhang Y. (2022). Improved Stability and Skin Penetration through Glycethosomes Loaded with Glycyrrhetinic Acid. Int. J. Cosmet. Sci..

[98] Sahoo R., Jacob P.J.S., Sahoo S. (2013). Biomedical Applications of Green Biopolymer Guar Gum. J. Pharm. Biomed. Sci..

[99] Chemical Book Guar Gum. https://www.chemicalbook.com/ChemicalProductProperty_EN_CB5253559.htm.

[100] Rodríguez-Hernández D., Demuner A.J., Barbosa L.C.A., Csuk R., Heller L. (2015). Hederagenin as a Triterpene Template for the Development of New Antitumor Compounds. Eur. J. Med. Chem..

[101] Nguyen L.T.H., Oh T.-W., Nguyen U.T., Choi M.-J., Yang I.-J., Shin H.-M. (2020). A Natural Compound Mixture Containing Arctigenin, Hederagenin, and Baicalein Alleviates Atopic Dermatitis in Mice by Regulating HPA Axis and Immune Activity. Evid.-Based Complement. Altern. Med..

[102] Rodríguez-Hernández D., Barbosa L.C.A., Demuner A.J., Nain-Perez A., Ferreira S.R., Fujiwara R.T., de Almeida R.M., Heller L., Csuk R. (2017). Leishmanicidal and Cytotoxic Activity of Hederagenin-Bistriazolyl Derivatives. Eur. J. Med. Chem..

[103] Zhang D., Sun J., Yang B., Ma S., Zhang C., Zhao G. (2020). Therapeutic Effect of Tetrapanax Papyriferus and Hederagenin on Chronic Neuropathic Pain of Chronic Constriction Injury of Sciatic Nerve Rats Based on KEGG Pathway Prediction and Experimental Verification. Evid.-Based Complement. Altern. Med..

[104] Gorgani L., Mohammadi M., Najafpour G.D., Nikzad M. (2017). Piperine—The Bioactive Compound of Black Pepper: From Isolation to Medicinal Formulations. Compr. Food Sci. Food Saf..

[105] Jung S.K., Choi D.W., Jung C.H., Kim Y., Jung S.Y. (2015). Piper Nigrum Fruit Extract Prevents TMA-Induced Allergic Contact Dermatitis by Regulating Th2 Cytokine Production. J. Agric. Sci..

[106] Choi D.W., Jung S.Y., Shon D.-H., Shin H.S. (2020). Piperine Ameliorates Trimellitic Anhydride-Induced Atopic Dermatitis-Like Symptoms by Suppressing Th2-Mediated Immune Responses via Inhibition of STAT6 Phosphorylation. Molecules.

[107] Politi S., Carvalho S.G., Rodero C.F., Pini K., Meneguin B., Sorrechia R., Chiavacci L.A., Chorilli M. (2023). International Journal of Biological Macromolecules Piperine-Loaded Nanoparticles Incorporated into Hyaluronic Acid/Sodium Alginate-Based Membranes for the Treatment of Inflammatory Skin Diseases. Int. J. Biol. Macromol..

[108] Yang D., Wang T., Long M., Li P. (2020). Quercetin: Its Main Pharmacological Activity and Potential Application in Clinical Medicine. Oxid. Med. Cell. Longev..

[109] Wadhwa K., Kadian V., Puri V., Bhardwaj B.Y., Sharma A., Pahwa R., Rao R., Gupta M., Singh I. (2022). New Insights into Quercetin Nanoformulations for Topical Delivery. Phytomed. Plus.

[110] Karuppagounder V., Arumugam S., Thandavarayan R.A., Sreedhar R., Giridharan V.V., Watanabe K. (2016). Molecular Targets of Quercetin with Anti-Inflammatory Properties in Atopic Dermatitis. Drug Discov. Today.

[111] Lee H.N., Shin S.A., Choo G.S., Kim H.J., Park Y.S., Kim B.S., Kim S.K., Cho S.D., Nam J.S., Choi C.S. (2018). Anti-Inflammatory Effect of Quercetin and Galangin in LPS-Stimulated RAW264.7 Macrophages and DNCB-Induced Atopic Dermatitis Animal Models. Int. J. Mol. Med..

[112] Beken B., Serttas R., Yazicioglu M., Turkekul K., Erdogan S. (2020). Quercetin Improves Inflammation, Oxidative Stress, and Impaired Wound Healing in Atopic Dermatitis Model of Human Keratinocytes. Pediatr. Allergy Immunol. Pulmonol..

[113] Salehi B., Sharopov F., Tumer T.B., Ozleyen A., Rodríguez-Pérez C., Ezzat S.M., Azzini E., Hosseinabadi T., Butnariu M., Sarac I. (2019). Symphytum Species: A Comprehensive Review on Chemical Composition, Food Applications and Phytopharmacology. Molecules.

[114] Wen S., Zhang J., Yang B., Elias P.M., Man M.Q. (2020). Role of Resveratrol in Regulating Cutaneous Functions. Evid.-Based Complement. Altern. Med..

[115] Ratz-Łyko A., Arct J. (2019). Resveratrol as an Active Ingredient for Cosmetic and Dermatological Applications: A Review. J. Cosmet. Laser Ther..

[116] Salehi B., Mishra A.P., Nigam M., Sener B., Kilic M., Sharifi-Rad M., Fokou P.V.T., Martins N., Sharifi-Rad J. (2018). Resveratrol: A Double-Edged Sword in Health Benefits. Biomedicines.

[117] Kang M.C., Cho K., Lee J.H., Subedi L., Yumnam S., Kim S.Y. (2019). Effect of Resveratrol-Enriched Rice on Skin Inflammation and Pruritus in the NC/Nga Mouse Model of Atopic Dermatitis. Int. J. Mol. Sci..

[118] Karuppagounder V., Arumugam S., Thandavarayan R.A., Pitchaimani V., Sreedhar R., Afrin R., Harima M., Suzuki H., Nomoto M., Miyashita S. (2014). Resveratrol Attenuates HMGB1 Signaling and Inflammation in House Dust Mite-Induced Atopic Dermatitis in Mice. Int. Immunopharmacol..

[119] Sozmen S.C., Karaman M., Micili S.C., Isik S., Ayyildiz Z.A., Bagriyanik A., Uzuner N., Karaman O. (2016). Resveratrol Ameliorates 2,4-Dinitrofluorobenzene-Induced Atopic Dermatitis-like Lesions through Effects on the Epithelium. PeerJ.

[120] Soldati P.P., Polonini H.C., Paes C.Q., Restrepob J.A.S., Creczynksi-Pasa T.B., Chaves M.G.A.M., Brandão M.A.F., Pittella F., Raposo N.R.B. (2018). Controlled Release of Resveratrol from Lipid Nanoparticles Improves Antioxidant Effect. IFAC-PapersOnLine.

[121] Sun R., Zhao G., Ni S., Xia Q. (2014). Lipid Based Nanocarriers with Different Lipid Compositions for Topical Delivery of Resveratrol: Comparative Analysis of Characteristics and Performance. J. Drug Deliv. Sci. Technol..

[122] Shrotriya S.N., Ranpise N.S., Vidhate B.V. (2017). Skin Targeting of Resveratrol Utilizing Solid Lipid Nanoparticle-Engrossed Gel for Chemically Induced Irritant Contact Dermatitis. Drug Deliv. Transl. Res..

[123] Ren S., Gao Y., Wang L., Qiu C., Yang L., Li L., Xiao Y., Xiao N., Liao L., Zuo Z. (2022). Sacran Polysaccharide Improves Atopic Dermatitis through Inhibiting Th2 Type Immune Response. Life Sci..

[124] Goto M., Azuma K., Arima H., Kaneko S., Higashi T., Motoyama K., Michihara A., Shimizu T., Kadowaki D., Maruyama T. (2021). Sacran, a Sulfated Polysaccharide, Suppresses the Absorption of Lipids and Modulates the Intestinal Flora in Non-Alcoholic Steatohepatitis Model Rats. Life Sci..

[125] Fukushima S., Motoyama K., Tanida Y., Higashi T., Ishitsuka Y., Kondo Y., Irie T., Tanaka T., Ihn H., Arima H. (2016). Clinical Evaluation of Novel Natural Polysaccharides Sacran as a Skincare Material for Atopic Dermatitis Patients. J. Cosmet. Dermatol. Sci. Appl..

[126] Bijak M. (2017). Silybin, a Major Bioactive Component of Milk Thistle (*Silybum Marianum* L. Gaernt.)—Chemistry, Bioavailability, and Metabolism. Molecules.

[127] Di Costanzo A., Angelico R. (2019). Formulation Strategies for Enhancing the Bioavailability of Silymarin: The State of the Art. Molecules.

[128] Rigon C., Marchiori M.C.L., da Silva Jardim F., Pegoraro N.S., Chaves P.D.S., Velho M.C., Beck R.C.R., Ourique A.F., Sari M.H.M., de Oliveira S.M. (2019). Hydrogel Containing Silibinin Nanocapsules Presents Effective Anti-Inflammatory Action in a Model of Irritant Contact Dermatitis in Mice. Eur. J. Pharm. Sci..

[129] Shrotriya S.N., Vidhate B.V., Shukla M.S. (2017). Formulation and Development of Silybin Loaded Solid Lipid Nanoparticle Enriched Gel for Irritant Contact Dermatitis. J. Drug Deliv. Sci. Technol..

[130] Mady F.M., Essa H., El-Ammawi T., Abdelkader H., Hussein A.K. (2016). Formulation and Clinical Evaluation of Silymarin Pluronic-Lecithin Organogels for Treatment of Atopic Dermatitis. Drug Des. Dev. Ther..

[131] Gao J., Zhang Y., Liu X., Wu X., Huang L., Gao W. (2021). Triptolide: Pharmacological Spectrum, Biosynthesis, Chemical Synthesis and Derivatives. Theranostics.

[132] Park K.S. (2021). Pharmacological Effects of *Centella asiatica* on Skin Diseases: Evidence and Possible Mechanisms. Evid.-Based Complement. Altern. Med..

[133] Susilawati Y., Chaerunisa A.Y., Purwaningsih H. (2021). Phytosome Drug Delivery System for Natural Cosmeceutical Compounds: Whitening Agent and Skin Antioxidant Agent. J. Adv. Pharm. Technol. Res..

[134] Ho P.J., Sung J.J., Cheon K.K., Tae H.J. (2018). Anti-Inflammatory Effect of *Centella asiatica* Phytosome in a Mouse Model of Phthalic Anhydride-Induced Atopic Dermatitis. Phytomedicine.

[135] He C., Xiao P. (2017). Origins, Phytochemistry, Pharmacology, Analytical Methods and Safety of Cortex Moutan (*Paeonia suffruticosa* Andrew): A Systematic Review. Molecules.

[136] Chung B., Chan L., Lun K., Hon E., Chung P., Wing S., Pui K., Yuk M., Lee H., Yung H. (2008). Traditional Chinese Medicine for Atopic Eczema: PentaHerbs Formula Suppresses Inflammatory Mediators Release from Mast Cells. J. Ethnopharmacol..

[137] Tsang M.S.M., Jiao D., Chan B.C.L., Hon K., Leung P.C., Lau C.B.S., Wong E.C.W., Cheng L., Chan C.K.M., Lam C.W.K. (2016). Atopic Dermatitis-Like Skin Inflammation. Molecules.

[138] Phan M.G., Do T.T., Nguyen T.N., Viet T., Do H., Dong N.P., Vu M.T. (2021). Chemical Constituents of Eupatorium Japonicum and Anti-Inflammatory, Cytotoxic, and Apoptotic Activities of Eupatoriopicrin on Cancer Stem Cells. Hindawi Evid.-Based Complement. Altern. Med. Hoechst.

[139] Shin J., Jeon Y., Lee S., Lee Y.G., Kim J.B., Kwon H.C., Kim S.H., Kim I., Lee K., Han Y.S. (2018). Apoptotic and Anti-Inflammatory Effects of Eupatorium Japonicum Thunb. in Rheumatoid Arthritis Fibroblast-Like Synoviocytes. BioMed Res. Int..

[140] Damiani G., Eggenhöffner R., Pigatto P.D.M., Bragazzi N.L. (2019). Nanotechnology Meets Atopic Dermatitis: Current Solutions, Challenges and Future Prospects. Insights and Implications from a Systematic Review of the Literature. Bioact. Mater..

[141] Touré A., Xueming X. (2010). Flaxseed Lignans: Source, Biosynthesis, Metabolism, Antioxidant Activity, Bio-Active Components, and Health Benefits. Compr. Rev. Food Sci. Food Saf..

[142] Hashempur M.H., Homayouni K., Ashraf A., Salehi A., Taghizadeh M., Heydari M. (2014). Effect of *Linum Usitatissimum* L. (Linseed) Oil on Mild and Moderate Carpal Tunnel Syndrome: A Randomized, Double-Blind, Placebo-Controlled Clinical Trial. DARU J. Pharm. Sci..

[143] James M.J., Gibson R.A., Cleland L.G. (2000). Dietary Polyunsaturated Fatty Acids and Inflammatory Mediator Production. Am. J. Clin. Nutr..

[144] Takic M., Pokimica B., Petrovic-Oggiano G., Popovic T. (2022). Effects of Dietary α-Linolenic Acid Treatment and the Efficiency of Its Conversion to Eicosapentaenoic and Docosahexaenoic Acids in Obesity and Related Diseases. Molecules.

[145] Thakur N., Garg G., Sharma P.K., Kumar N. (2012). Nanoemulsions: A Review on Various Pharmaceutical Application. Glob. J. Pharmacol..

[146] Shaban N.Z., Mohammed A.S., Abu-Serie M.M., Maher A.M., Habashy N.H. (2022). Inhibition of Oxidative Stress, IL-13, and WNT/β-Catenin in Ovalbumin-Sensitized Rats by a Novel Organogel of *Punica granatum* Seed Oil Saponifiable Fraction. Biomed. Pharmacother..

[147] Shaban N.Z., Talaat I.M., Elrashidy F.H., Hegazy A.Y., Sultan A.S. (2017). Therapeutic Role of *Punica granatum* (Pomegranate) Seed Oil Extract on Bone Turnover and Resorption Induced in Ovariectomized Rats. J. Nutr. Health Aging.

[148] Park D.K., Lee Y.G., Park H.J. (2013). Extract of Rhus Verniciflua Bark Sup- Presses 2,4-Dinitrofluorobenzene-Induced Allergic Contact Dermati-Tis. Evid.-Based Complement. Altern. Med..

[149] Jiang P., Sun H. (2018). Sulfuretin Alleviates Atopic Dermatitis-like Symptoms in Mice via Suppressing Th2 Cell Activity. Immunol. Res..

[150] Lam N.S., Long X., Su X.Z., Lu F. (2020). Melaleuca Alternifolia (Tea Tree) Oil and Its Monoterpene Constituents in Treating Protozoan and Helminthic Infections. Biomed. Pharmacother..

